# Supplementary material for: 7D, a small molecule inhibits dengue infection by increasing interferons and neutralizing-antibodies via CXCL4:CXCR3:p38:IRF3 and Sirt1:STAT3 axes respectively
Source: EMBO Mol Med. 2024 Sep 16;16(10):2376–401. doi: 10.1038/s44321-024-00137-8 (PMC11473809; doi:10.1038/s44321-024-00137-8)
Supplement: Supplementary file 1 — Appendix [file 44321_2024_137_MOESM1_ESM.pdf]

7D, a small molecule inhibits dengue infection by increasing interferons and neutralizing-antibodies via CXCL4:CXCR3:p38:IRF3 and Sirt1:STAT3 axes respectively

Kishan Kumar Gaur[1,#], Tejeswara Rao Asuru[1,#], Mitul Srivastava[2], Nitu Singh[1], Nikil Purushotham[3], Boja Poojary[3], Bhabatosh Das[2], Sankar Bhattacharyya[2,$,], Shailendra Asthana [2, $,*] and Prasenjit Guchhait[1,$,*]

[1]Regional Centre for Biotechnology, National Capital Region Biotech Science Cluster, Faridabad, India;
[2]Translational Health Science Technology Institute, National Capital Region Biotech Science Cluster, Faridabad, India;
[3]Department of Studies in Chemistry, Mangalore University, Mangalagangotri, Karnataka, India.
[#] First authors with equal contribution, [$]Senior authors;
[*]Correspondence at prasenjit@rcb.res.in, and sasthana@thsti.res.in

Supplementary materials:

Appendix Figure S1: Strategy for generation of mouse-adapted DENV2 (P23085 INDI-60), and standardizing infection mouse models. (Page 2)

Appendix Figure S2: In vivo cytotoxicity data of compound 7D. (Page 3)

Appendix Figure S3: CXCR3$^{-/-}$ and WT mice infected with DENV2. (Page 4)

Appendix Figure S4: Gating strategy to immunophenotyping including P-STAT3/ Ac-STAT3 status positive plasma cells. (Page 5)

Appendix Figure S5: Anti- dengue IgM and IgG indirect elisa scatter plots. (Page 6)

Appendix Figure S6: WT mice BMDM's data related to data in Figure EV6. (Page 7)

Appendix Figure S7: Infection of human monocytes with DENV2 (P23085 INDI-60 strain). (Page 8)

Appendix Tables S1: Nucleotide substitution in mouse-adapted DENV2 genome. (Page 9)

Appendix Tables S2: List of primers. (Page 9)

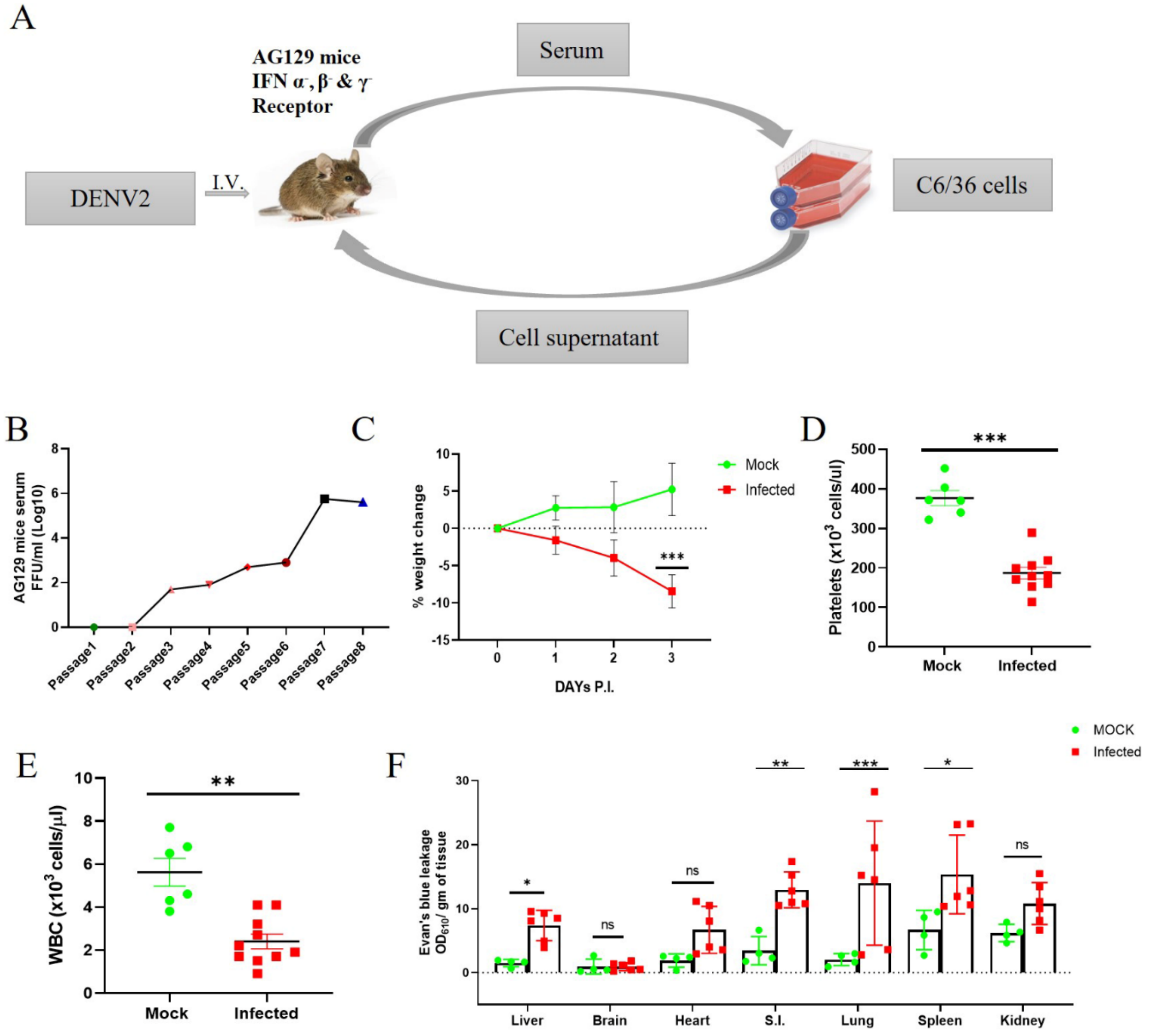

Appendix Figure S1: (A) Schematic for generation of mouse-adapted DENV2 (P23085 INDI-60): alternate passage strategy was used. Serum from DENV2-infected AG129 mice was cultured in mosquito C636 cell line in vitro. DENV2 isolated from C636 cells was injected in AG129 mice. This alternate passage continued for 8 times to generate mouse adopted- DENV2. **(B)** DENV2 was quantified by FFU assay in 8 passages. **(C)** Change in the body weight is recorded for AG129 mice infected with mouse adapted DENV2 strain (P8-P23085INDI-60), n=2 mock, n=8 infected mice, Data are mean ± SEM. Two-way ANOVA was used, ***P<0.001 (*P* values: 0.0008).. **(D-E)** Platelet, n=6 mock, n=10 infected mice, (*P* values: 0.001) and WBC counts were measured from whole blood of mice from the above experiment, using hematology analyzer, n=6 mock, n=10 infected mice, Data are median ± IQR. Mann-Whitney U test was used, **P<0.01, ***P<0.001 (*P* values: 0.005). **(F)** Vascular leakage assay: Evan's blue dye extravasation in different organs of DENV2-infected AG129 mice n=4 mock, n=6 infected mice, Data mean ± SEM. *P<0.05, **P<0.01, ***P<0.001, ns=non-significant (*P*

values: 0.04; 0.003; 0.0002; 0.01). The nucleotide substitution of the DENV2 genome is mentioned in table-S1.

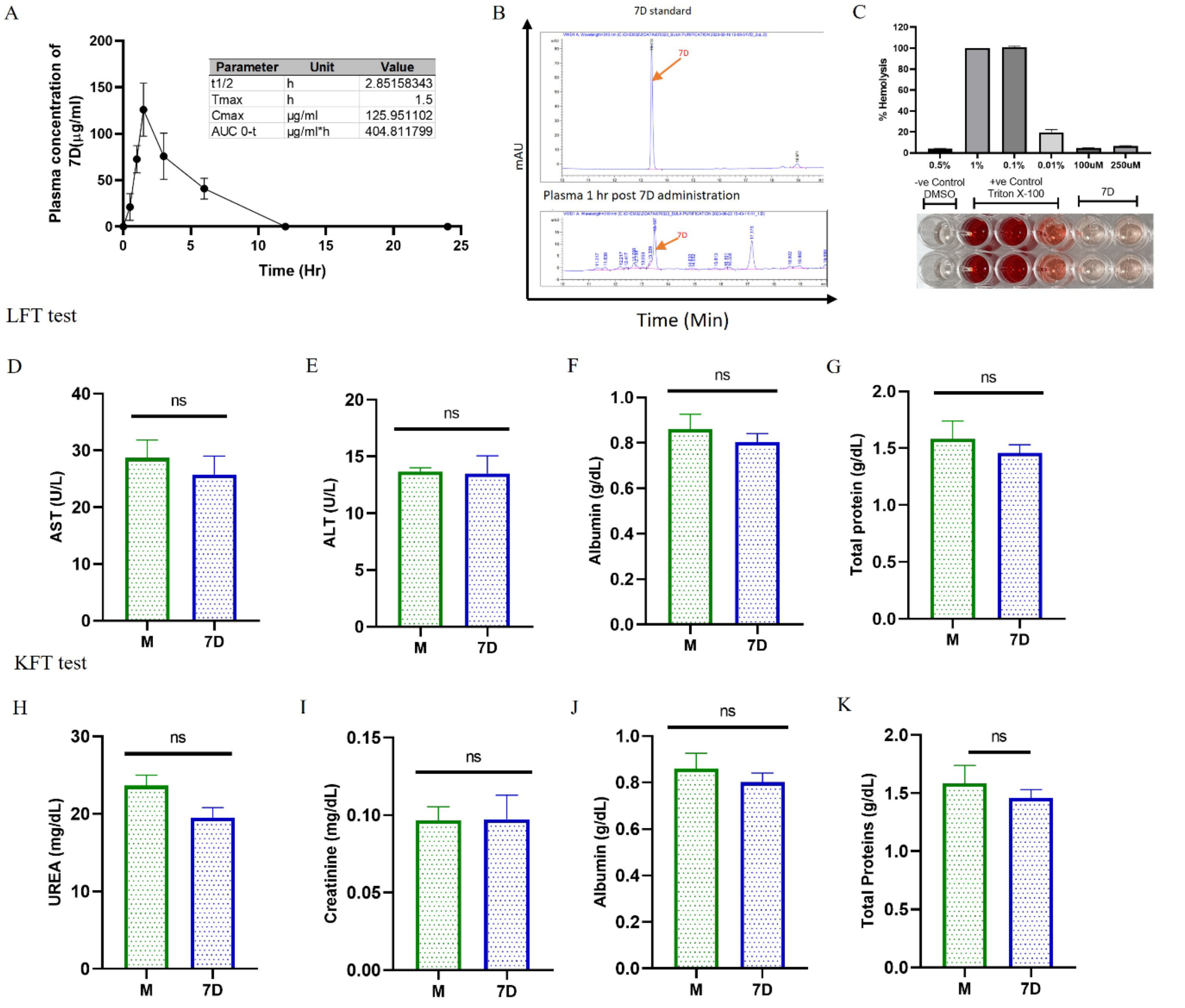

Appendix Figure S2: (A) Plasma concentration of 7D was measured in mice plasma using HPLC after single dose injection of the compound (8 mg/kg body weight). $T_{1/2}$, C_{max} ~2.85 was measured from HPLC data at different time points to determine the AUC using PKSolver, n=5. **(B)** 7D peak in HPLC chromatograms. Effect of 7D on **(C)** RBC lysis (hemolysis), n=3 independent experiments, and **(D to G)** Liver and **(H to K)** Kidney function was tested in mice. 7D (8 mg/kg body weight dose/day) was administered for four days n=3 mock, n=4 7d. Data are median ± IQR. Mann-Whitney U test was used, ns=non-significant.

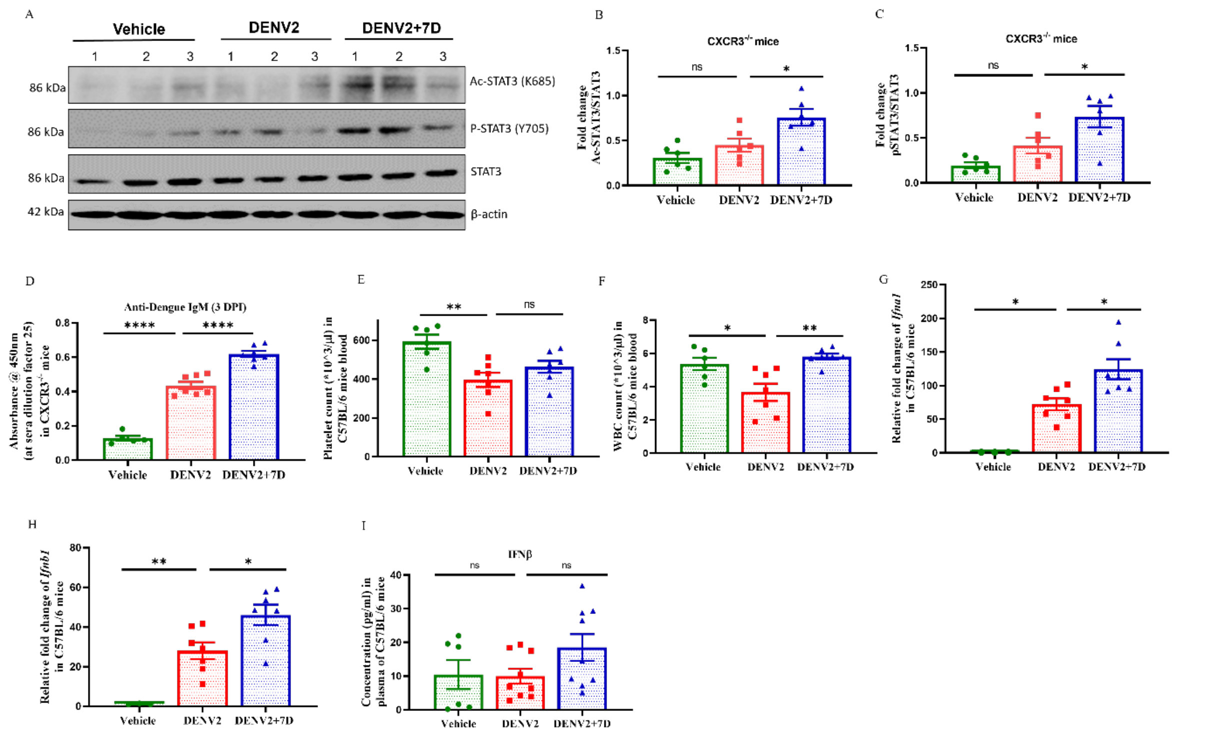

Appendix Figure S3: Data related to Figure 6, **(A-C)** Western blot analysis of Ac-STAT3, P-STAT3 and STAT3 from the spleenocytes of CXCR3$^{-/-}$ mice n=6 mice per group. **(D)** Anti-dengue IgM level was measured in plasma of CXCR3$^{-/-}$ mice n=5 vehicle, n=7 mice per group. **(E)** Platelets and **(F)** WBC counts were taken in whole blood using hematology analyzer n=6 vehicle, n=7 mice per group. Relative gene expression of **(G)** *ifna1* (IFNα) and, **(H)** *ifnb1* (IFNβ) were quantified in the spleen n=3 vehicle, n=7 mice per group and, **(I)** IFNβ levels were measured in plasma of WT mice n=6 vehicle, n=9 mice per group. (B-I) One-way ANOVA and Bonferroni's post-test were used. Data represented as mean ± SEM, *P<0.05, **P<0.01, ***P<0.001, ****P<0.0001 and ns=non-significant. (*P* values: B:0.02, C:0.04, D: 0.0001; 0.0001, E:0.0019, F:0.01; 0.0017, G:0.02; 0.01 and H:0.008; 0.02).

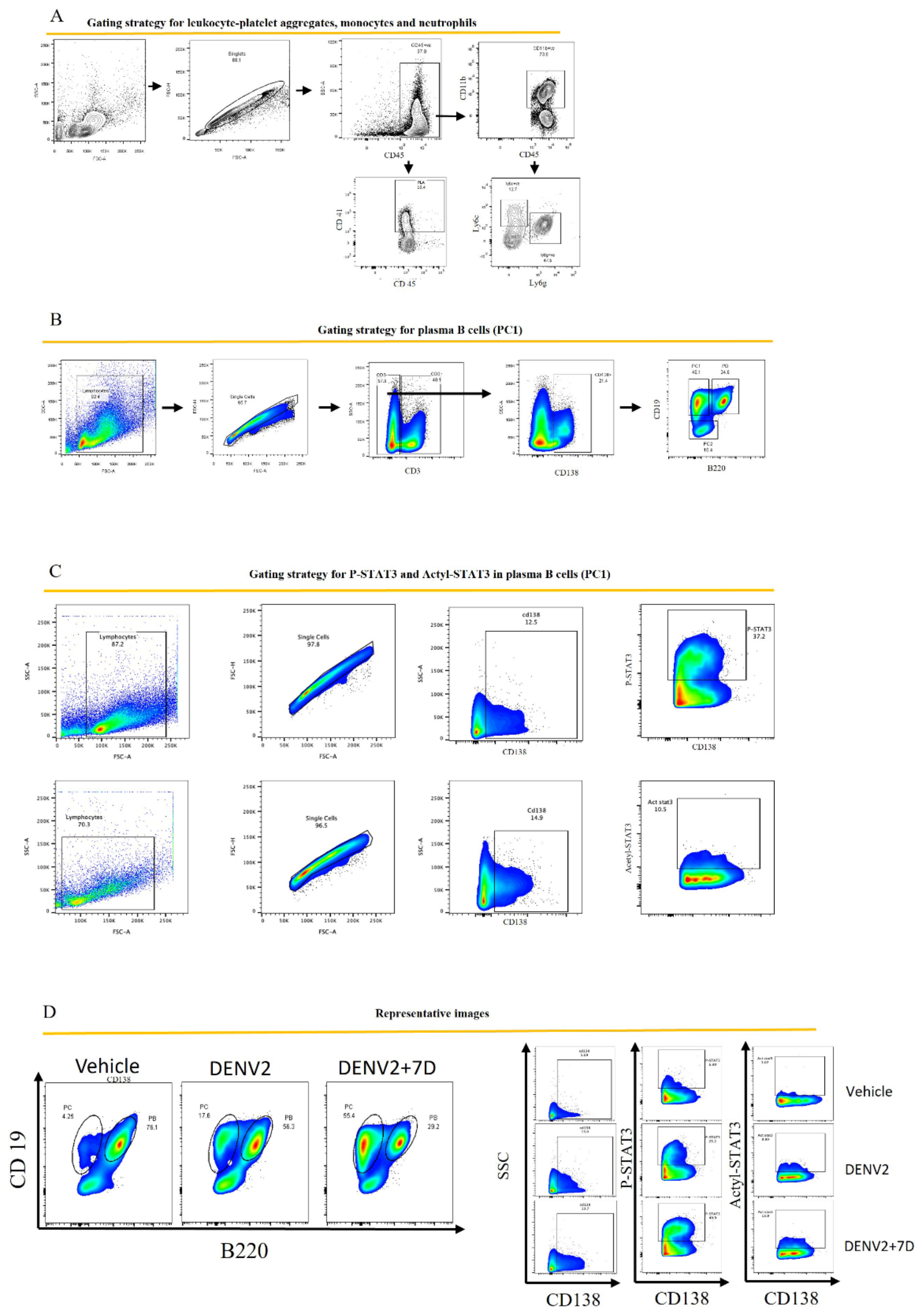

Appendix Figure S4: (A) Gating strategy followed for platelet leukocyte aggregates, monocytes, and neutrophil counts of mice. **(B)** B-cell gating strategy to identify plasma B-cells. **(C)** B-cell gating strategy to measure P-STAT3 and Ac-STAT3 status in plasma B-cells. **(D)** Representative images for Fig 5 E, H, I and J.

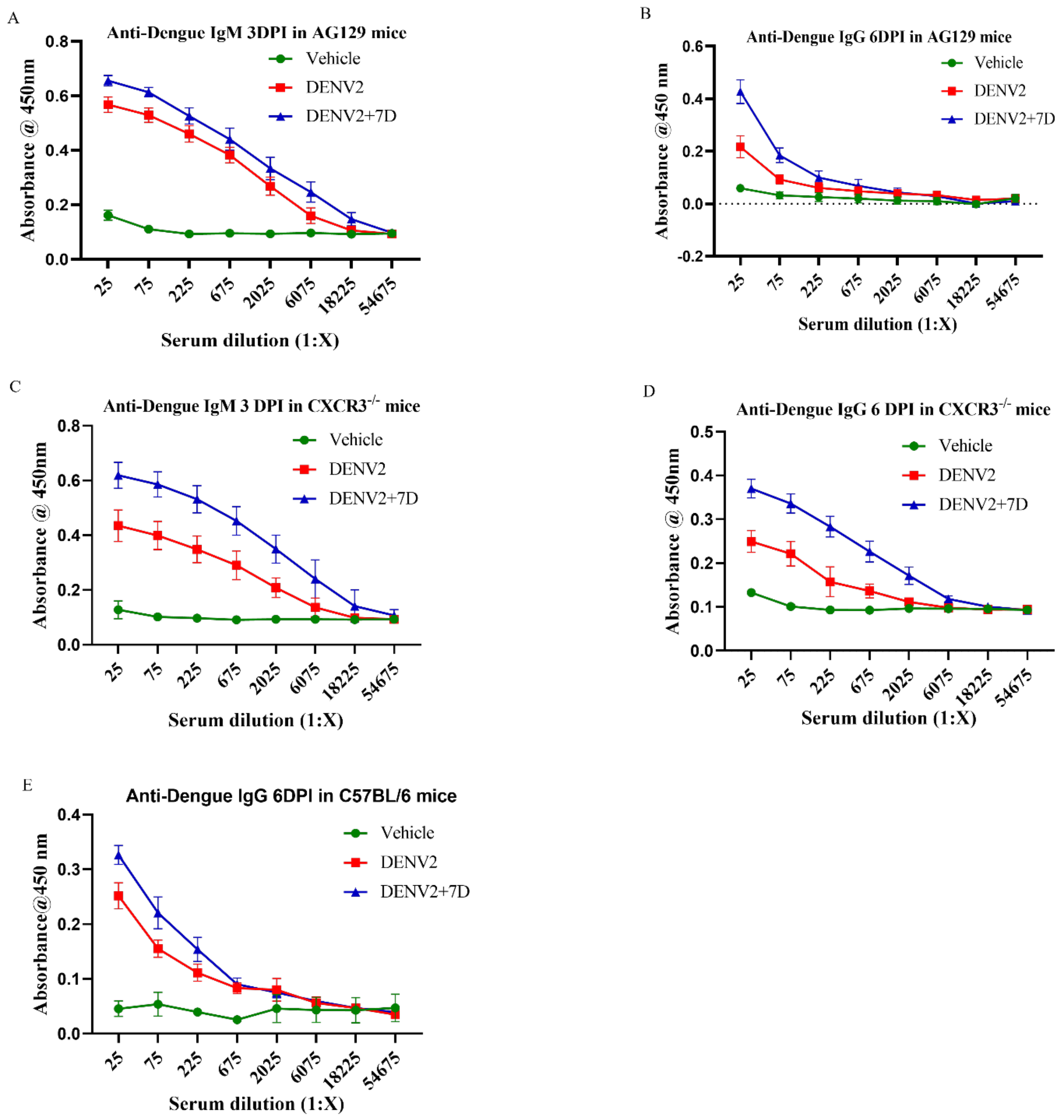

Appendix Figure S5: (A-E) Mean OD_{450} values and serum dilutions of dengue specific IgM and IgG in the serum of dengue infected AG129 mice, WT and $CXCR3^{-/-}$ mice. Data information: (A-E) Data are mean ± SEM.

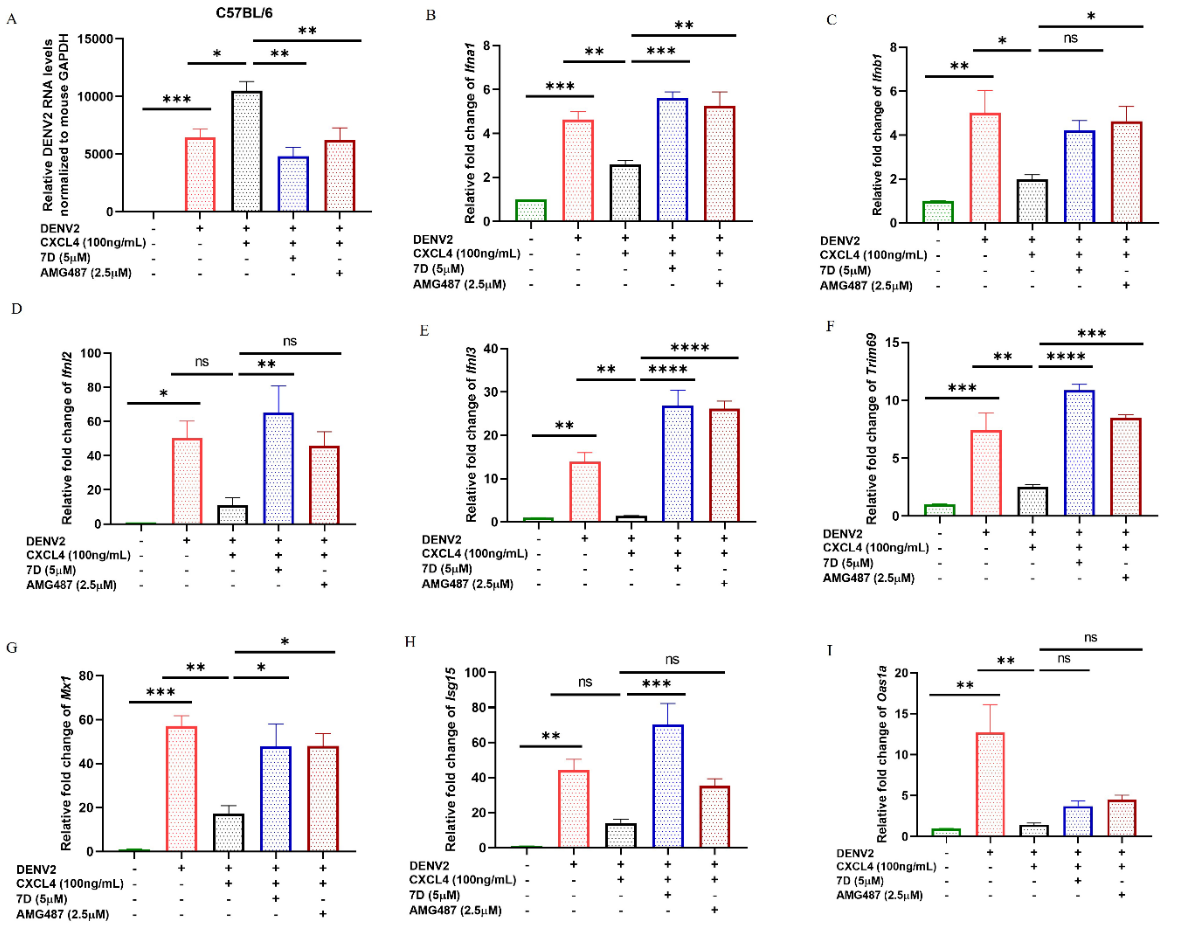

Appendix Figure S6: The bone marrow derived macrophages (BMDMs) isolated from C57BL/6 mice. **(A)** BMDMs infected with DENV2 (MOI~1) in presence of CXCL4 and treated with 7D and AMG487. DENV2 genome was quantified from 3 independent experiments by qRT-PCR. **(B-I)** Relative gene expression of type I, type III interferons and Interferon stimulatory genes (ISGs) were quantified from 3 independent experiment mentioned above. **(B)** Ifna1 (IFNα), **(C)** Ifnb1 (IFNβ), **(D)** Ifnl2 (IFNλ28a), **(E)** Ifnl3 (IFNλ28b), **(F)** Trim69, **(G)** Mx1, **(H)** Isg15 and, **(I)** Oas1a. (A-I) One-way ANOVA and Bonferroni's post-test were used. Data represented as mean ± SEM, *P<0.05, **P<0.01, ***P<0.001, ****P<0.0001 and ns=non-significant (*P* values: A:0.0005; 0.01; 0.002; 0.009, B:0.001; 0.009; 0.0005; 0.0015, C:0.002; 0.01; 0.03, D:0.01; 0.008, E:0.004; 0.005; 0.0001; 0.0001, F:0.0004; 0.003; 0.0001; 0.0007, G:0.0002; 0.002; 0.01; 0.01, H:0.003; 0.0004 and I 0.0013; 0.0018).

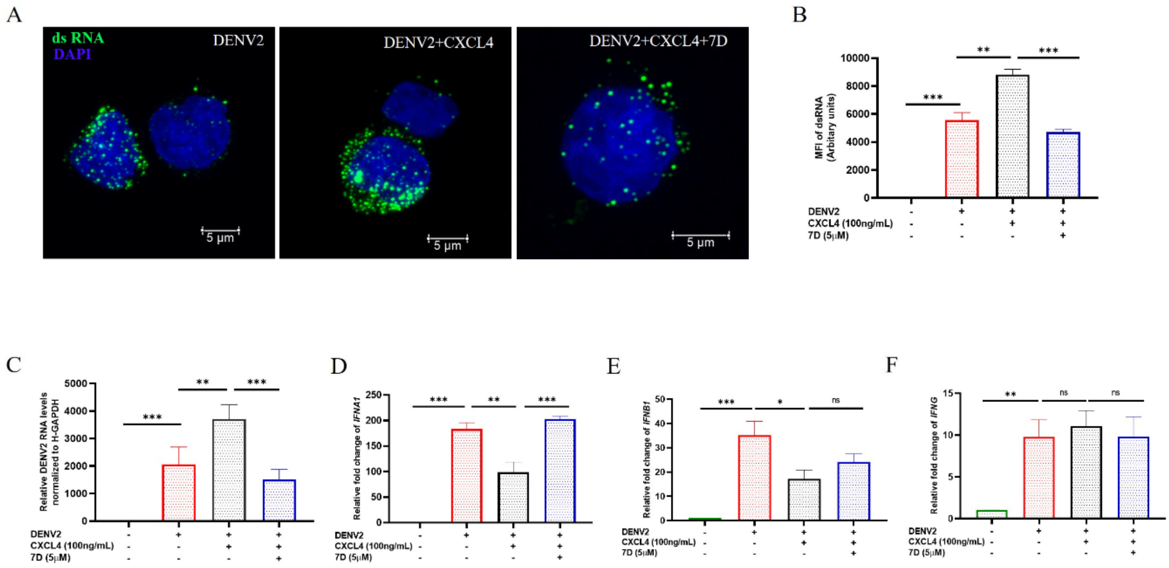

Appendix Figure S7: Human monocytes infected with DENV2 (P23085 INDI-60) with MOI~3 and treated with 5uM 7D for 24 hr. **(A and B)** Cells were stained for dsRNA and analyzed using microscopy. Data are mean ± SEM MFI from three individual experiments, Kruskal-Wallis test is used to compared the groups, *P<0.05, **P<0.01, ***P<0.001(*P* values: 0.001; 0.002; 0.001). **(C)** DENV2 genome was quantified from above experiment using qRT-PCR n=3 independent experiments. **(D - F)** Relative expression of *IFNA1*, *IFNB1* and *IFNG* (IFNα, β and γ) was measured using qRT-PCR n=3 independent experiments. (C-F) One-way ANOVA and Bonferroni's post-test were used. Data are mean ± SEM, *P<0.05, **P<0.01, ***P<0.001 and ns=non-significant. (*P* values: C:0.0002; 0.0015; 0.001, D:0.001; 0.003; 0.0009 and E:0.0007; 0.03 and F 0.002).

Appendix Tables S1: Nucleotide substitution in DENV2 genome following adaption in AG129 mice. In amino acids, Only the substitutions are mentioned, and others remain unaltered.

Table S1. Nucleotide substitution in mouse adapted DENV2 (strain P8- P23085 INDI-60).

			Nucleotide		
Sr. no.	Position in RNA	Region in Polyprotein	DV2-P0	DV2-P8	Amino acid Substitution
1.	704	PrM	A	G	E203G
2.	1318	Envelope	A	G	K408E
3.	1851	Envelope	G	K	
4.	2868	NS1	T	C	
5.	3933	NS2A	T	Y	
6.	4466	NS2B	T	C	
7.	7105	NS4A	C	Y	
8.	7144	NS4A	C	M	V1457A
9.	9387	NS5	C	Y	

Appendix Tables S2: Primer sets for various genes used in this study.

Table S2. Primers used in this study.

**Human (Gene)**	**Forward primer (5’-3’)**	**Reverse primer (5’-3’)**
GAPDH	GCCACATCGCTCAGACACCAT	ACCAGGCGCCCAATACG
IFNA1	TGGGCTGTGATCTGCCTCAAAC	CAGCCTTTTGGAACTGGTTGCC
IFNB1	TGGCAATTGAATGGGAGGCT	TCATAGATGGTCACTGCGGC
IFNG	GAATGTCCAACGCAAAGCAA	TCCTTGTTCGCTTCCCTGTTT
Trim69	CCAACCCCTCCTCCAACATC	TAGCATCAGTGGGTCTCGGA
**Mouse (Gene)**		
GAPDH	ACCACAGTCCATGCCATCAC	TCCACCACCCTGTTGCTGTA
Ifna1	ATGGCTAGRCTCTGTGCTTTCCT	AGGGCTCTCCAGAYTTCTGCTCTG
Ifnb1	GCACTGGGTGGAATGAGACTATTG	TTCTGAGGCATCAACTGACAGGTC
Ifnl2	CCAGTGGAAGCAAAGGATTGCC	TCAGGTCCTTCTCAAGCAGCCT
Ifnl3	CCAGTGGAAGCAAAGGATTGCC	GCACCTCATGTCCTTCTCAAGC
Trim69	CACGGATGGAACAGCAGAACTC	CTGGATAGGACCTTTGAAGCGG
Oas1a	GAGGTGGAGTTTGATGTGCTGC	GTGAAGCAGGTAGAGAACTCGC
Mx1	TGGACATTGCTACCACAGAGGC	TTGCCTTCAGCACCTCTGTCCA
Isg15	TTGCCTTCAGCACCTCTGTCCA	CTCAGCCAGAACTGGTCTTCGT
**Dengue virus serotypes**		
DENV1	CAATGGATGACAACAGAAGAYATG	TCCATCCATGGGTTTTCCTCTAT
DENV2	GCAGAAACACAACATGGAACGATAGT	TGATGTAGCTGTCTCCGAATGG
DENV3	ATGGAATGTGTGTGGGAGGTGG	GGCTTTCTATCCARTAGCCCATG
DENV4	GCAGATCTCTGGAAAAATGAACCA	GAGAATCTCTTCACCAACCCGTG
**CXCR3$^{-/-}$ mice Genotyping**		
Mut Forward1	GGGCCAGCTCATTCCTCCCACTCAT	CGTGCACTATGCTCAGATATCTGT
WT Forward2	CCACAGGATTTCAGCCTGAACTTTG	
**AG129 mice Genotyping**		
Ifna1/b1r$^{-/-}$	ATTATTAAAAGAAAAGACGAGGCGAAGTGG	AAGATGTGCTGTTCCCTTCCTCTGCTCTGA
Ifngr$^{-/-}$	GATCCTACATACGAAACATACGGTC	GTCATCATGGAAAGGAAAGGAGGGATACAG
Neo Reverse		CCTGCGTGCAATCCATCTTG
**DENV2 sequencing**		
D2F1	AGTWGTTAGTCTACGTGGAC	TGGGCTGTCTTTTTCTGTGA
D2F2	GCAGAAACACAACATGGAACA	AACGCGTCAGTCAGTTCAAG
D2F3	GAAAGCTGACCTCCAAGGAA	CTGAAATGTCTGTCGTGACCA
D2F4	AGGCAGCTGGGATTTTCATGA	TTTCCCTTCTGGTGTGACCA
D2F5	ACTCAAGTATTGATGATGAGGA	TGTGTCCAATCGTTCCATCCT
D2F12	GCAGGATGGGACACAAGAAT	AGAACCTGTTGATTCAACAG
**DENV2 cDNA primer**	5’AGAACCTGTTGATTCAACAGCACC 3’	
