# Supplementary figures and images for: 7D, a small molecule inhibits dengue infection by increasing interferons and neutralizing-antibodies via CXCL4:CXCR3:p38:IRF3 and Sirt1:STAT3 axes respectively
Source: EMBO Mol Med. 2024 Sep 16;16(10):2376–401. doi: 10.1038/s44321-024-00137-8 (PMC11473809; doi:10.1038/s44321-024-00137-8)

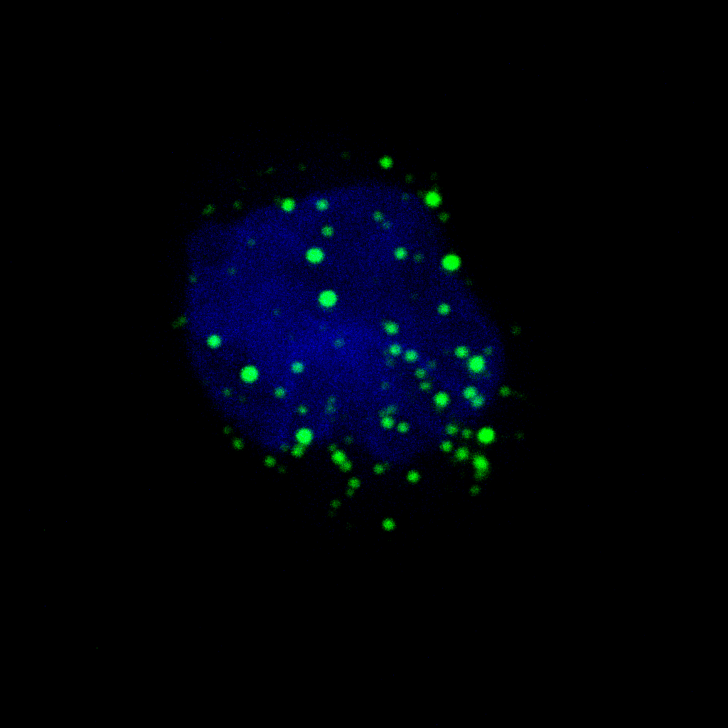

Supplement: Supplementary file 3 — Source data Fig. 1 [file 44321_2024_137_MOESM3_ESM.zip › Fig 1/Fig 1H/Microscopy images/DENV2.tif]

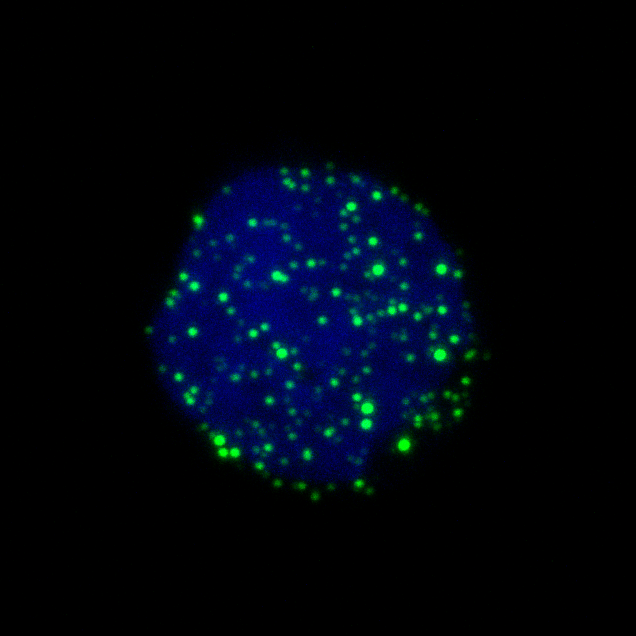

Supplement: Supplementary file 3 — Source data Fig. 1 [file 44321_2024_137_MOESM3_ESM.zip › Fig 1/Fig 1H/Microscopy images/DENV2+CXCL4.tif]

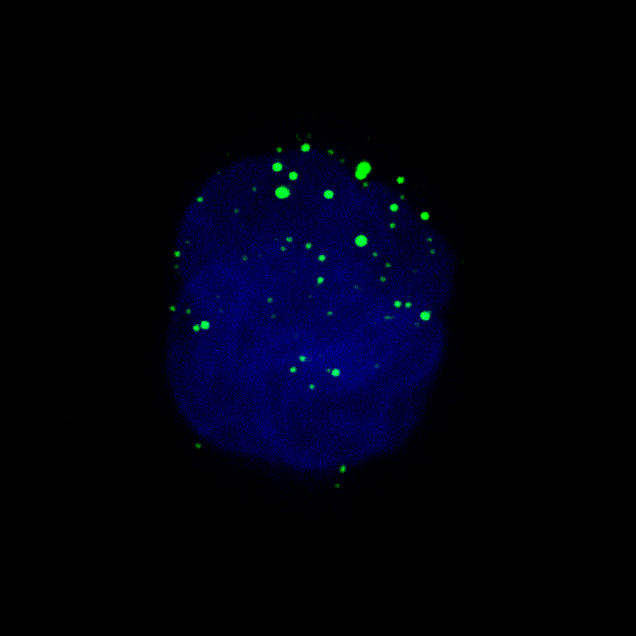

Supplement: Supplementary file 3 — Source data Fig. 1 [file 44321_2024_137_MOESM3_ESM.zip › Fig 1/Fig 1H/Microscopy images/DENV2+CXCL4+7D.tif]

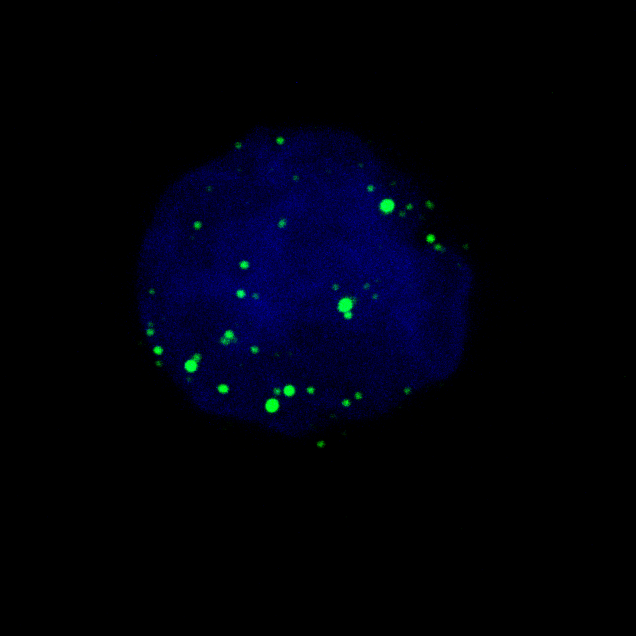

Supplement: Supplementary file 3 — Source data Fig. 1 [file 44321_2024_137_MOESM3_ESM.zip › Fig 1/Fig 1H/Microscopy images/DENV2+CXCL4+AMG487.tif]

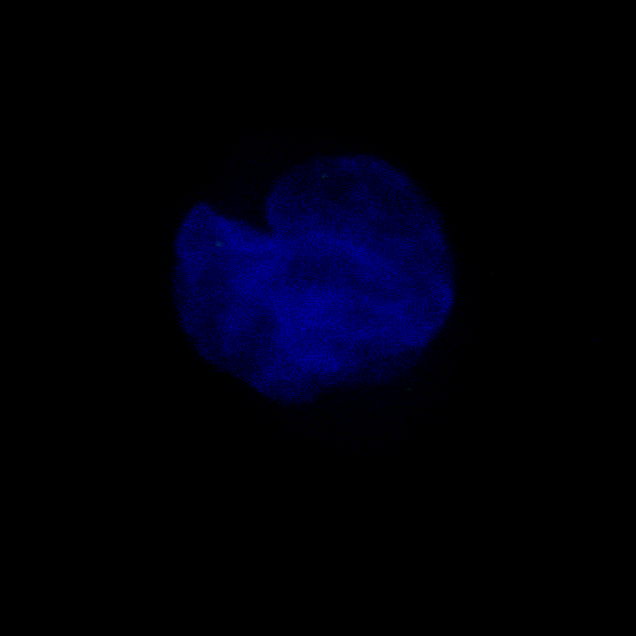

Supplement: Supplementary file 3 — Source data Fig. 1 [file 44321_2024_137_MOESM3_ESM.zip › Fig 1/Fig 1H/Microscopy images/MOCK.tif]

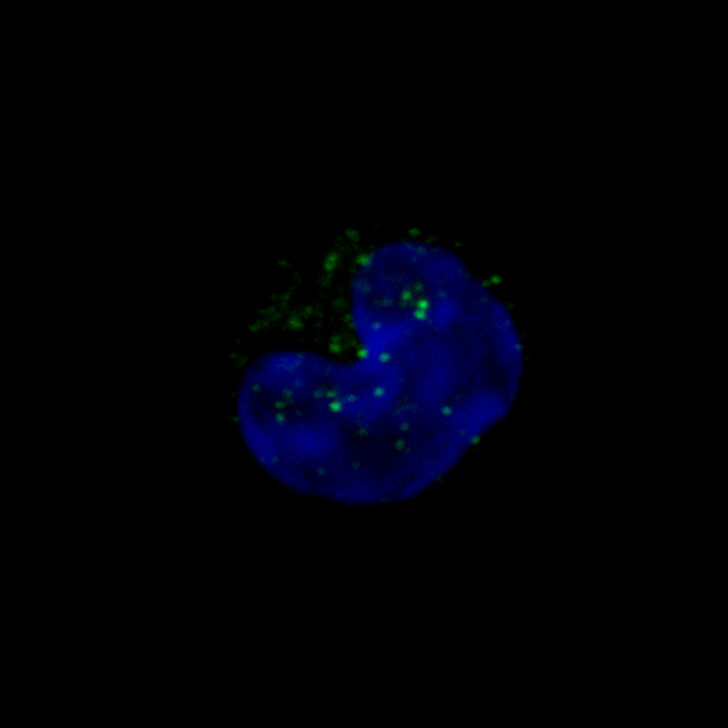

Supplement: Supplementary file 4 — Source data Fig. 2 [file 44321_2024_137_MOESM4_ESM.zip › Fig 2/Fig 2A/CXCR3 KO mice/Microscopy images/DENV2 + CXCL4.tif]

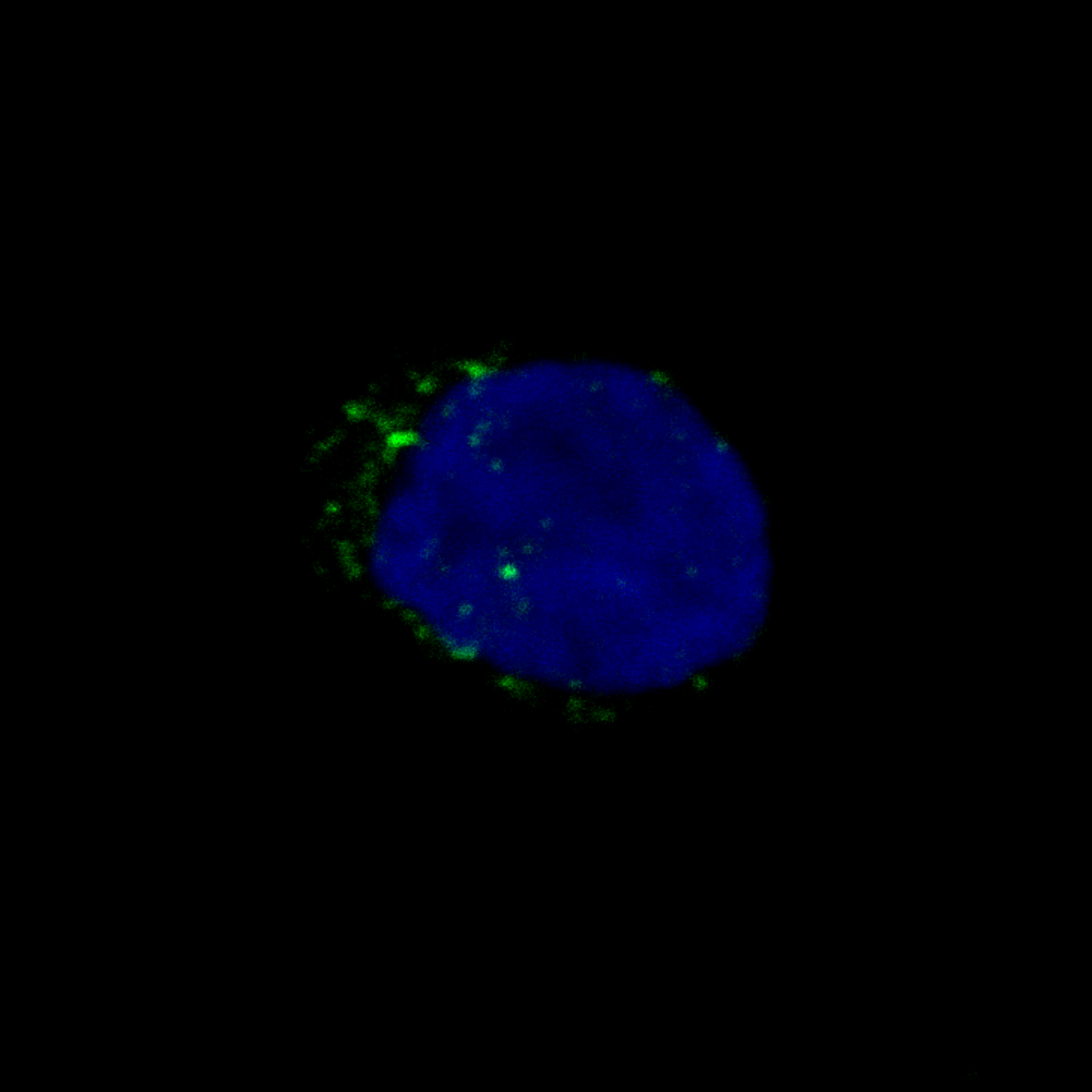

Supplement: Supplementary file 4 — Source data Fig. 2 [file 44321_2024_137_MOESM4_ESM.zip › Fig 2/Fig 2A/CXCR3 KO mice/Microscopy images/DENV2.tif]

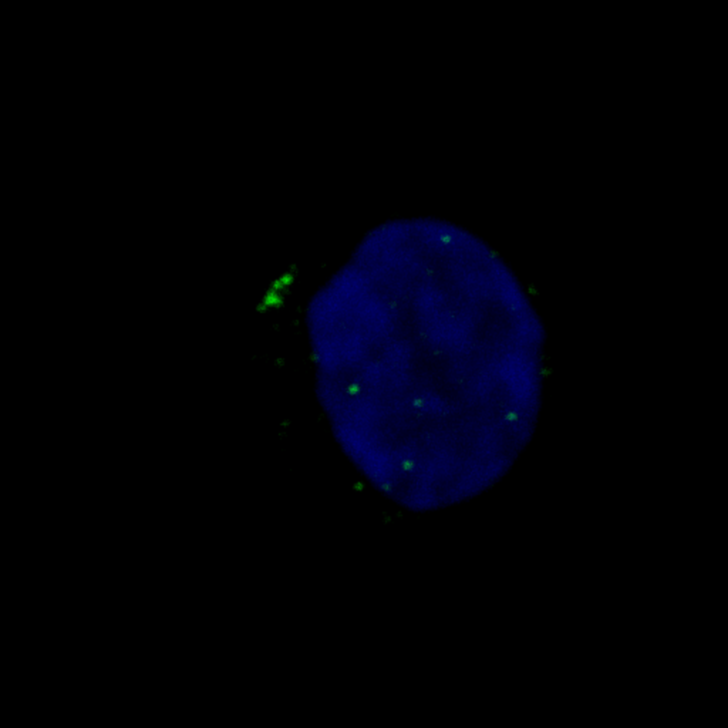

Supplement: Supplementary file 4 — Source data Fig. 2 [file 44321_2024_137_MOESM4_ESM.zip › Fig 2/Fig 2A/CXCR3 KO mice/Microscopy images/DENV2+CXCL4+7D.tif]

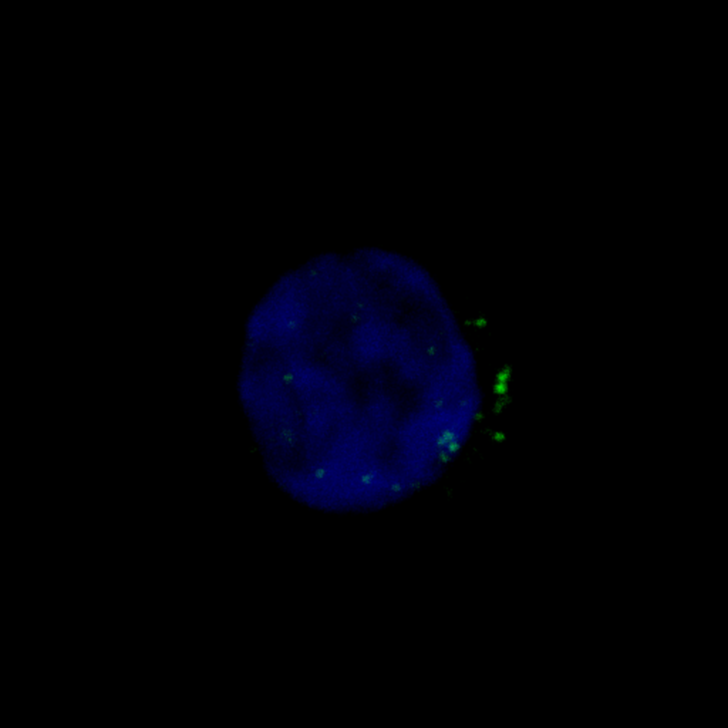

Supplement: Supplementary file 4 — Source data Fig. 2 [file 44321_2024_137_MOESM4_ESM.zip › Fig 2/Fig 2A/CXCR3 KO mice/Microscopy images/DENV2+CXCL4+AMG487.tif]

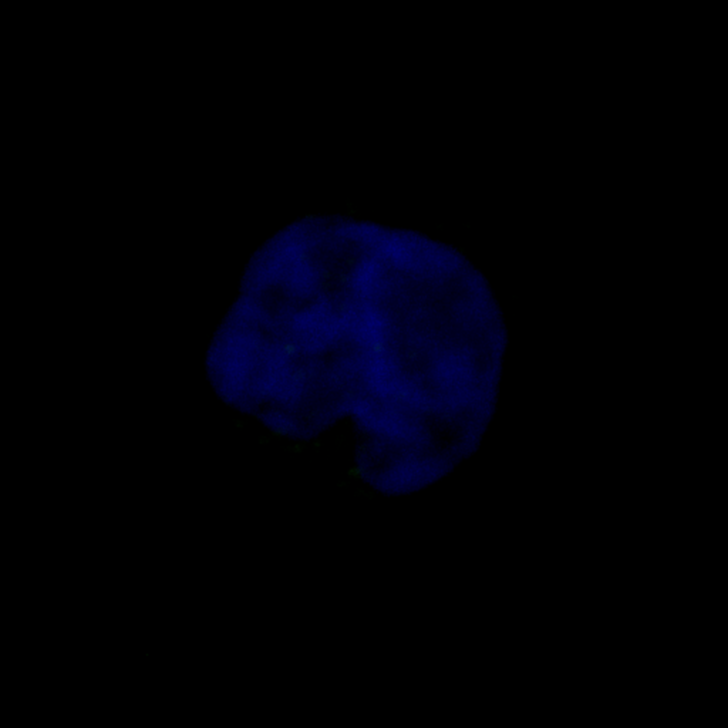

Supplement: Supplementary file 4 — Source data Fig. 2 [file 44321_2024_137_MOESM4_ESM.zip › Fig 2/Fig 2A/CXCR3 KO mice/Microscopy images/MOCK.tif]

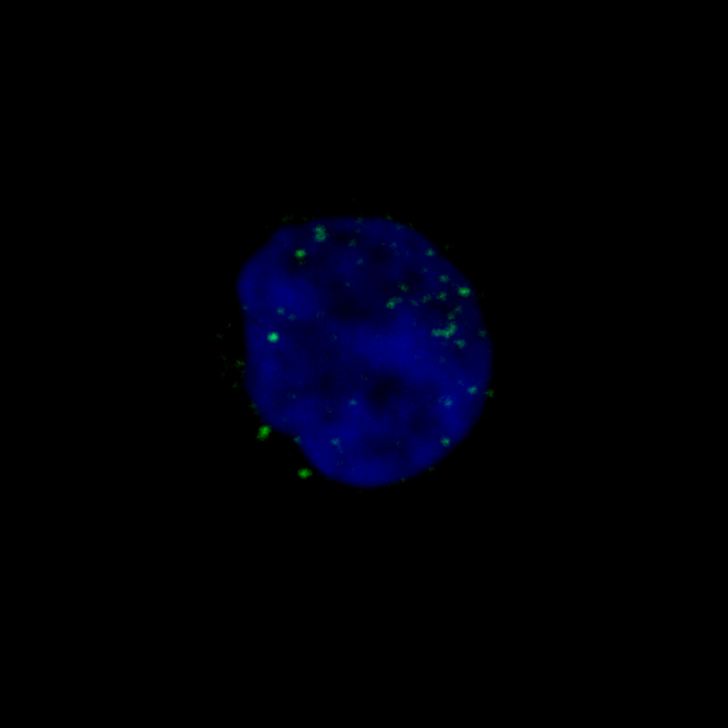

Supplement: Supplementary file 4 — Source data Fig. 2 [file 44321_2024_137_MOESM4_ESM.zip › Fig 2/Fig 2A/WT mice/Microscopy images/DENV2.tif]

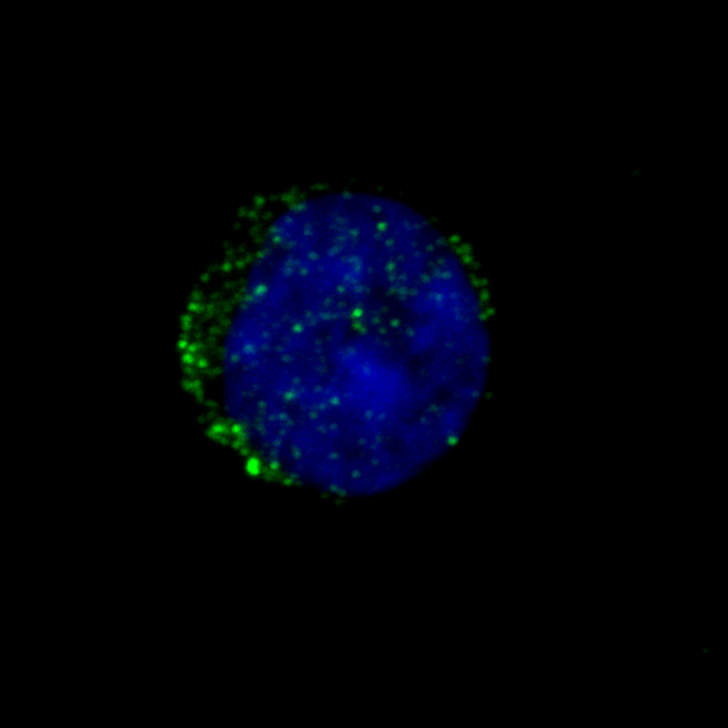

Supplement: Supplementary file 4 — Source data Fig. 2 [file 44321_2024_137_MOESM4_ESM.zip › Fig 2/Fig 2A/WT mice/Microscopy images/DENV2+CXCL4.tif]

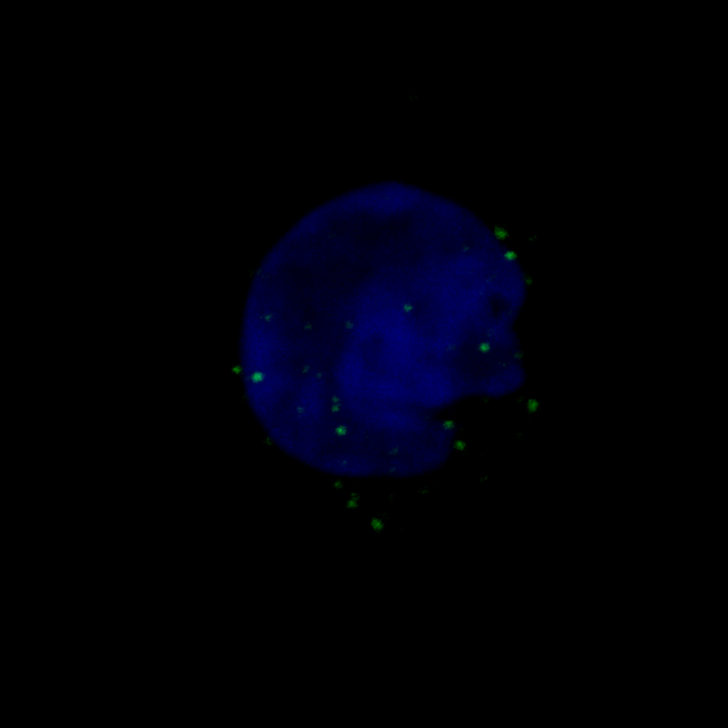

Supplement: Supplementary file 4 — Source data Fig. 2 [file 44321_2024_137_MOESM4_ESM.zip › Fig 2/Fig 2A/WT mice/Microscopy images/DENV2+CXCL4+7D.tif]

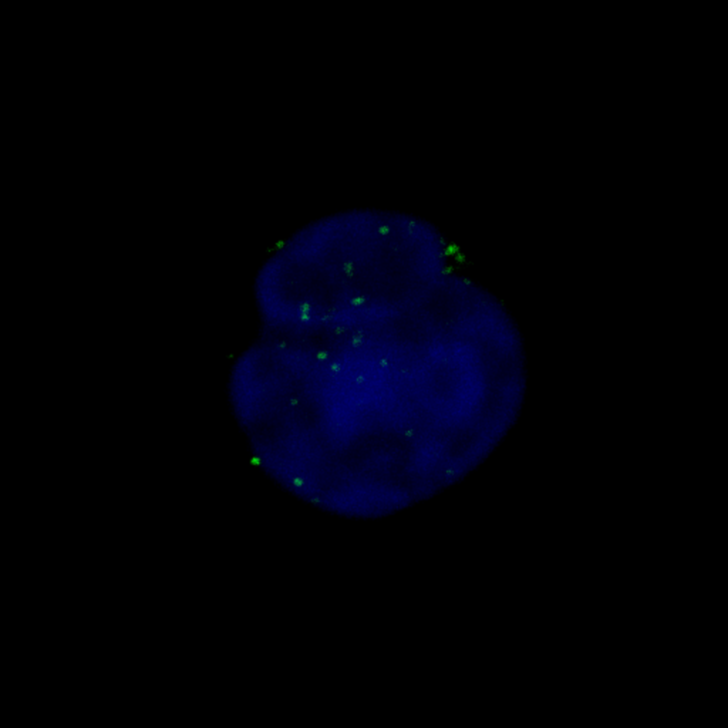

Supplement: Supplementary file 4 — Source data Fig. 2 [file 44321_2024_137_MOESM4_ESM.zip › Fig 2/Fig 2A/WT mice/Microscopy images/DENV2+CXCL4+AMG487.tif]

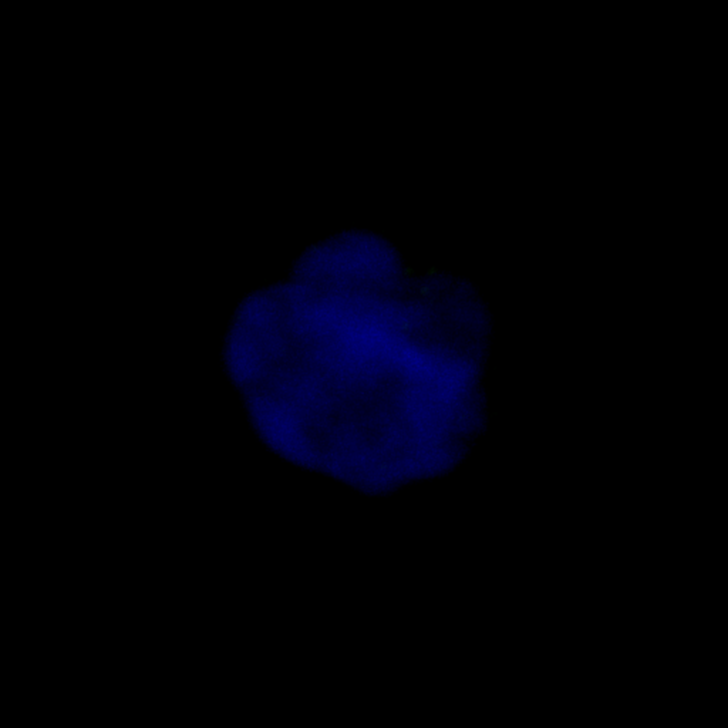

Supplement: Supplementary file 4 — Source data Fig. 2 [file 44321_2024_137_MOESM4_ESM.zip › Fig 2/Fig 2A/WT mice/Microscopy images/MOCK.tif]

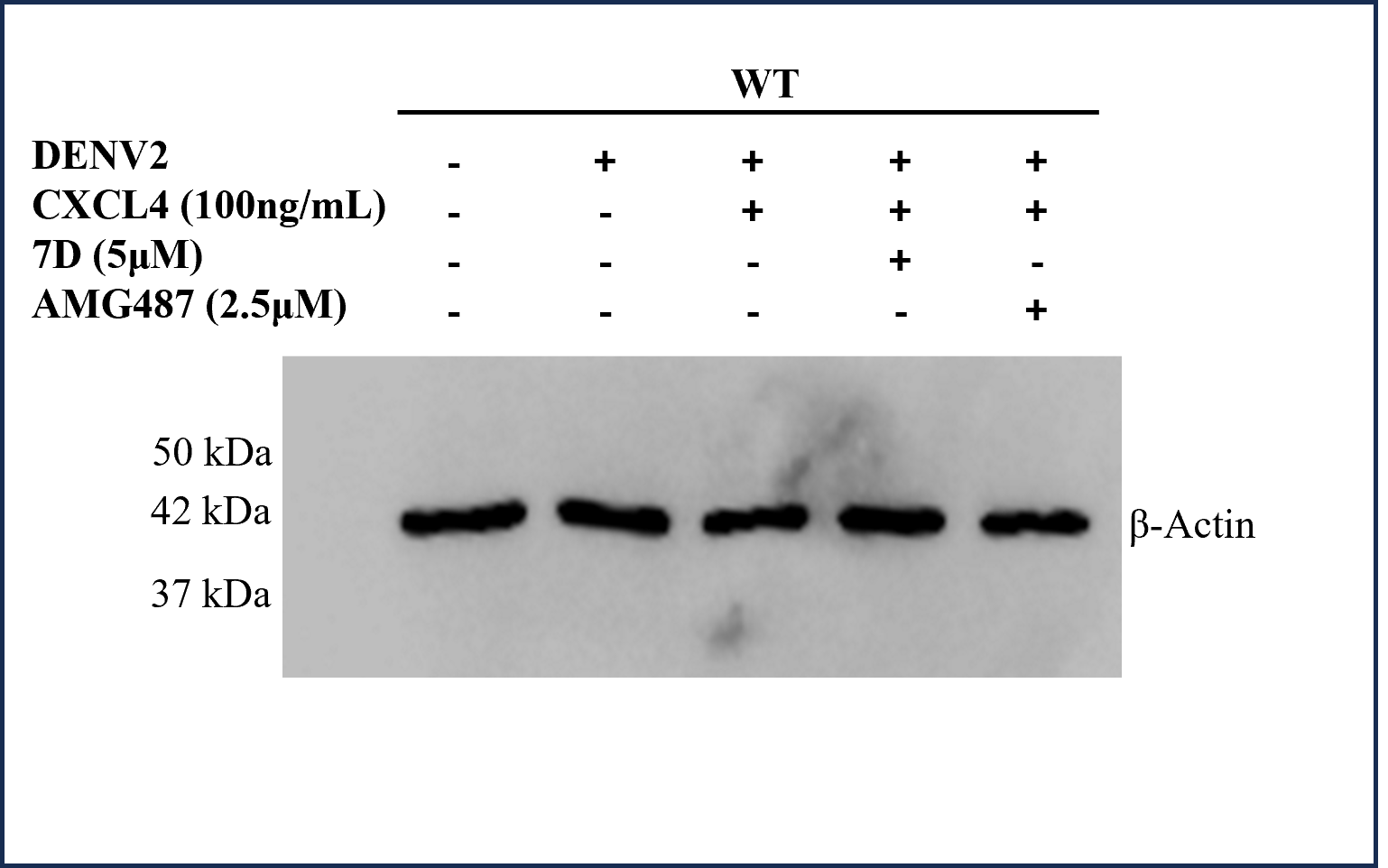

Supplement: Supplementary file 4 — Source data Fig. 2 [file 44321_2024_137_MOESM4_ESM.zip › Fig 2/Fig 2F/Western B-Actin.tif]

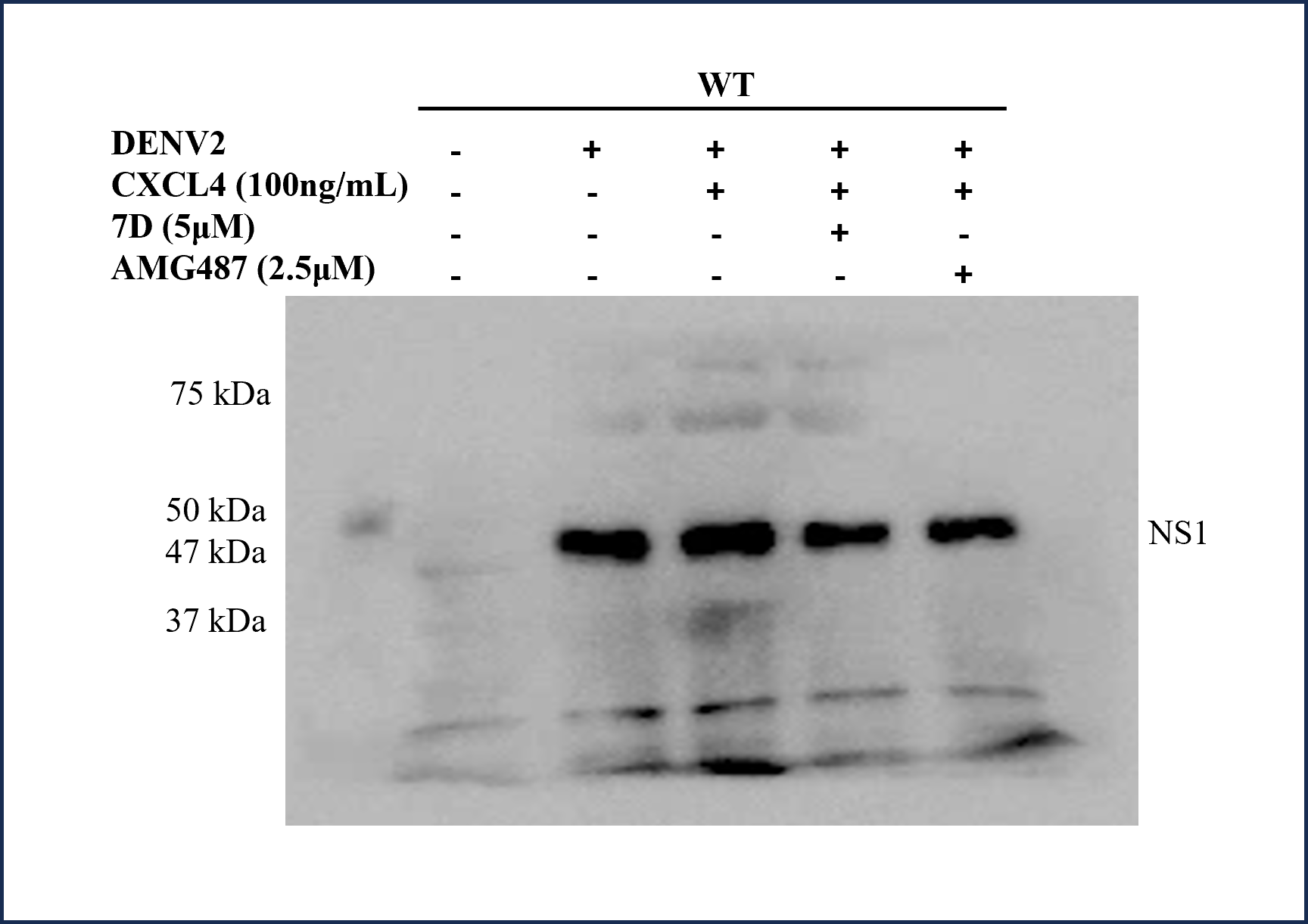

Supplement: Supplementary file 4 — Source data Fig. 2 [file 44321_2024_137_MOESM4_ESM.zip › Fig 2/Fig 2F/Western NS1.tif]

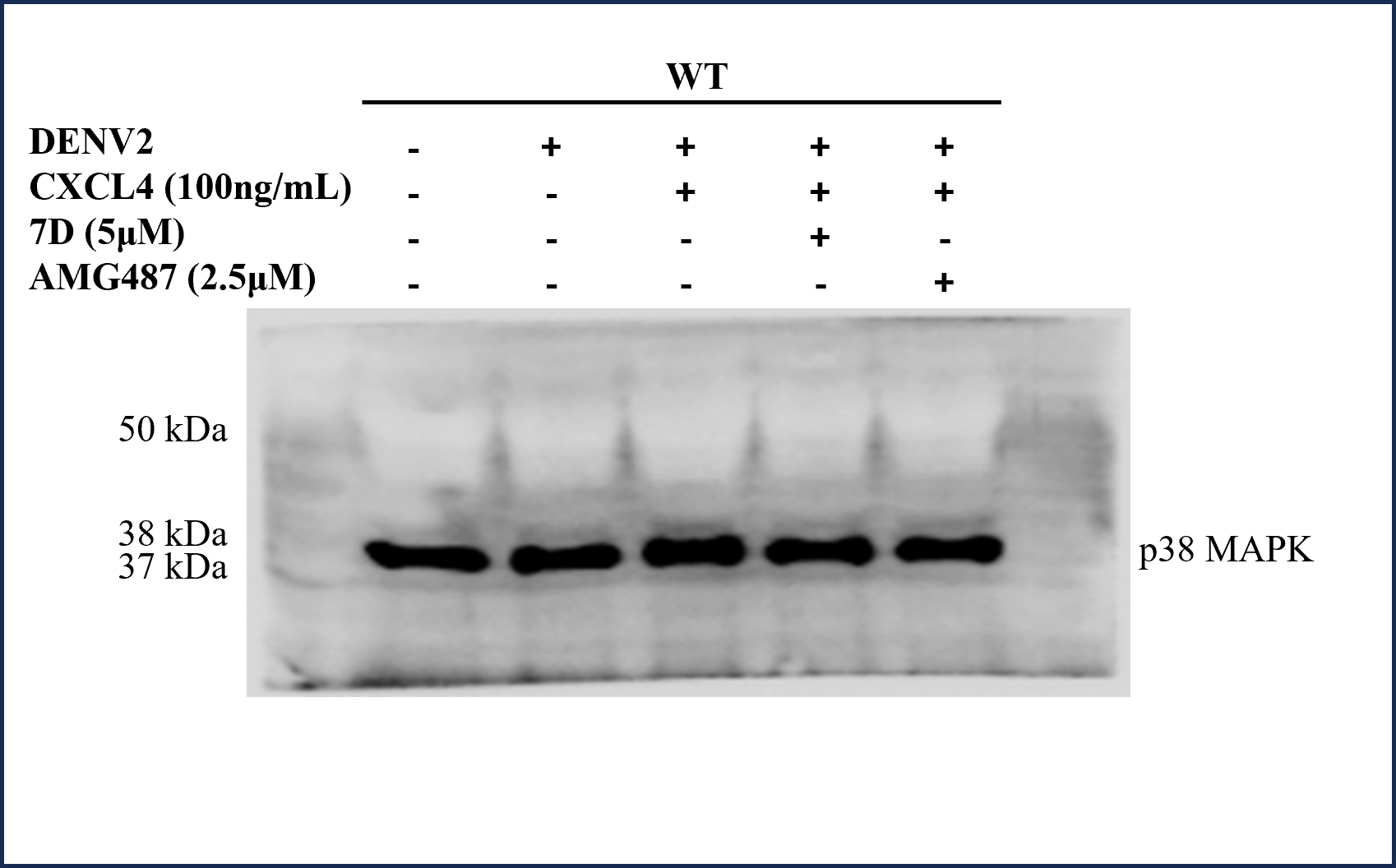

Supplement: Supplementary file 4 — Source data Fig. 2 [file 44321_2024_137_MOESM4_ESM.zip › Fig 2/Fig 2F/Western p38 MAPK.tif]

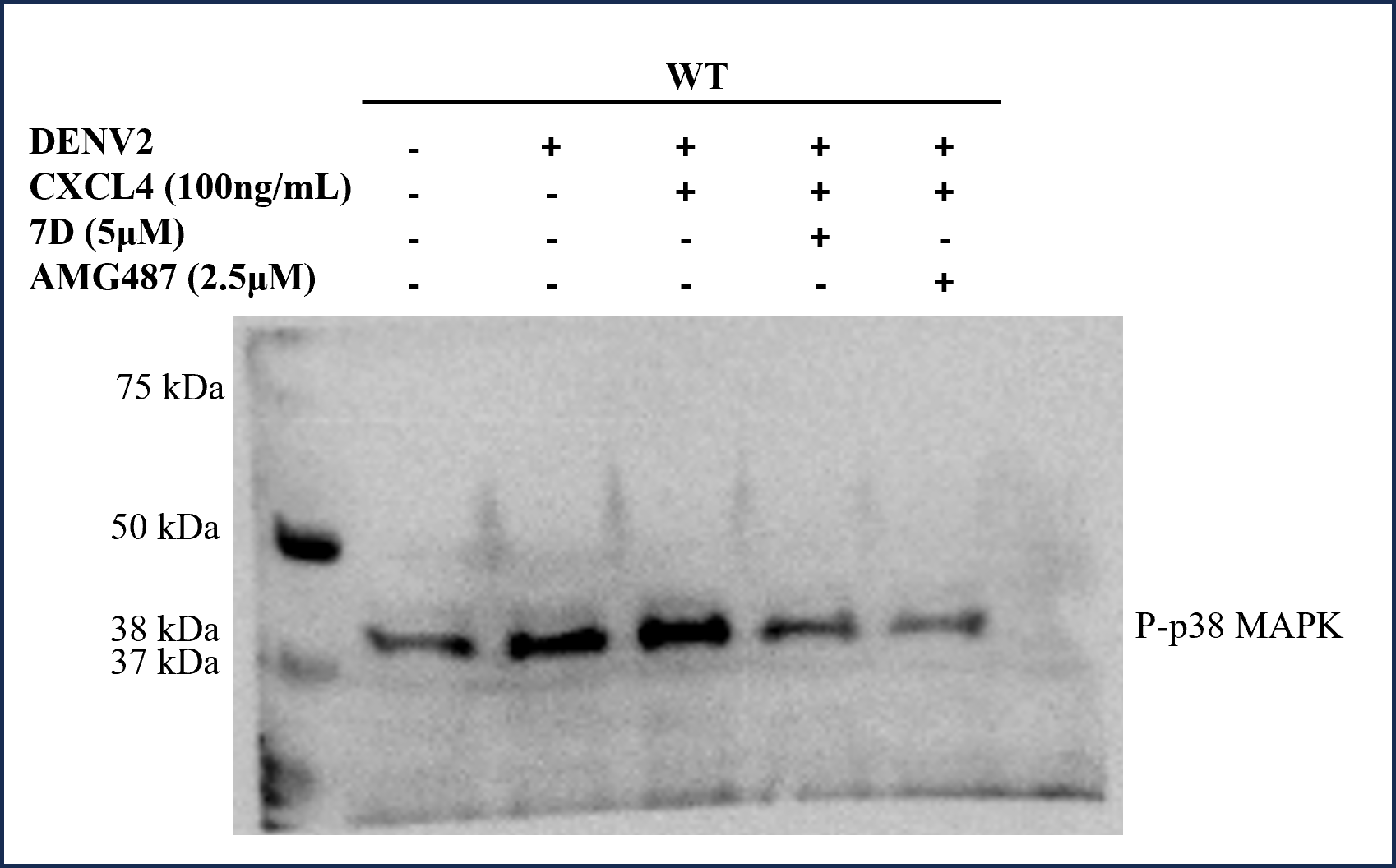

Supplement: Supplementary file 4 — Source data Fig. 2 [file 44321_2024_137_MOESM4_ESM.zip › Fig 2/Fig 2F/Western P-p38 MAPK.tif]

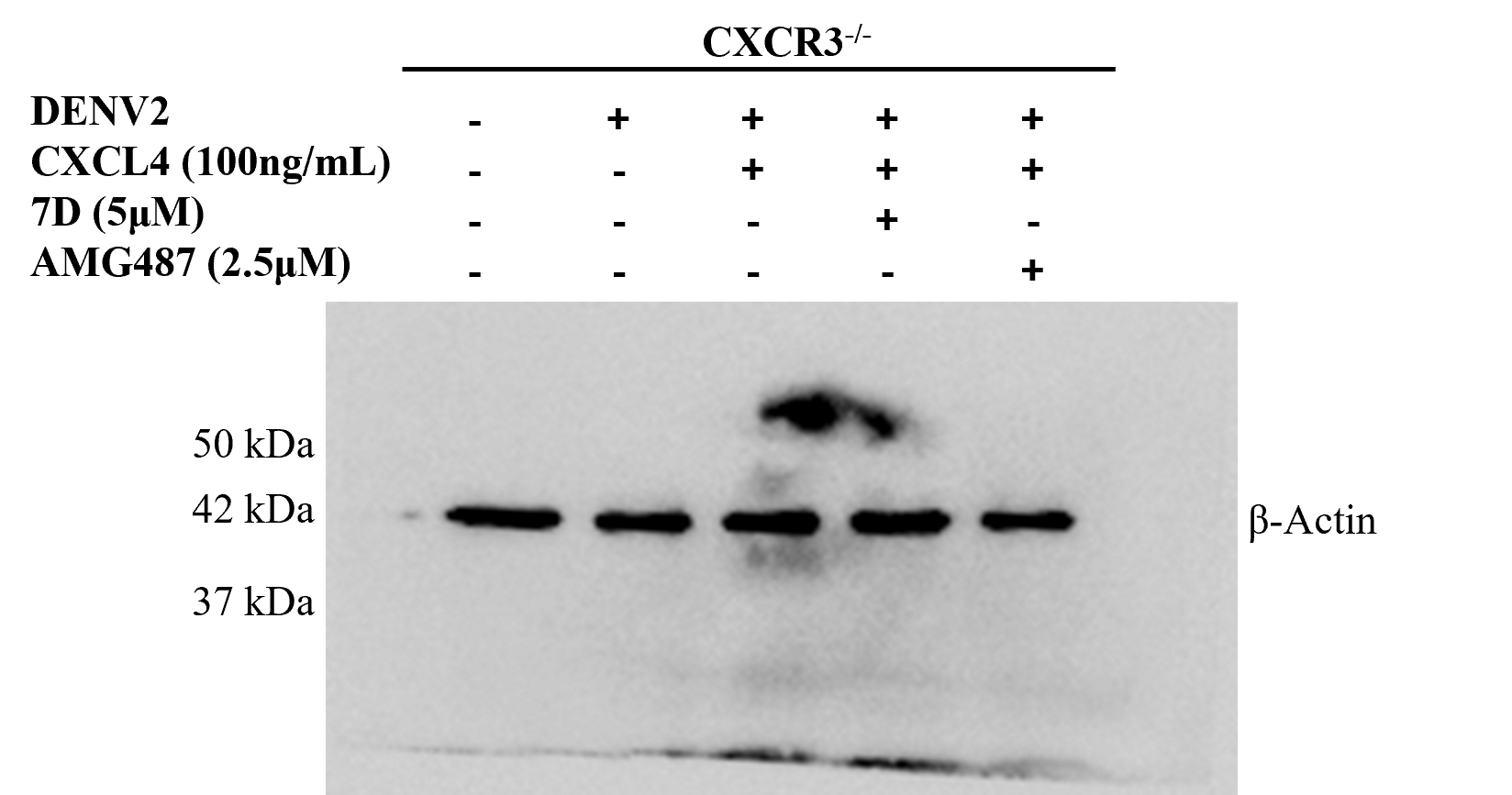

Supplement: Supplementary file 4 — Source data Fig. 2 [file 44321_2024_137_MOESM4_ESM.zip › Fig 2/Fig 2G/Western B-Actin.tif]

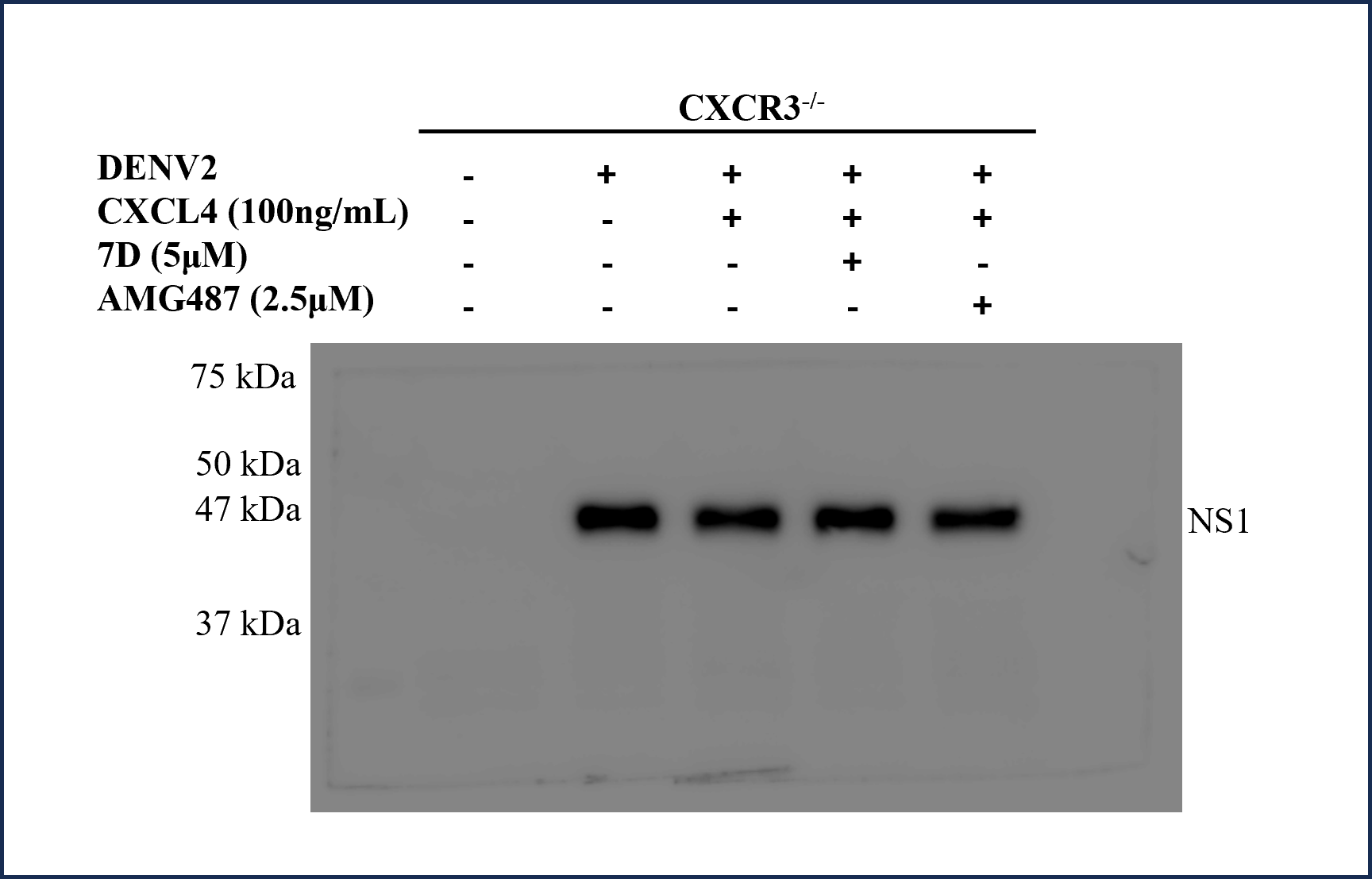

Supplement: Supplementary file 4 — Source data Fig. 2 [file 44321_2024_137_MOESM4_ESM.zip › Fig 2/Fig 2G/Western NS1.tif]

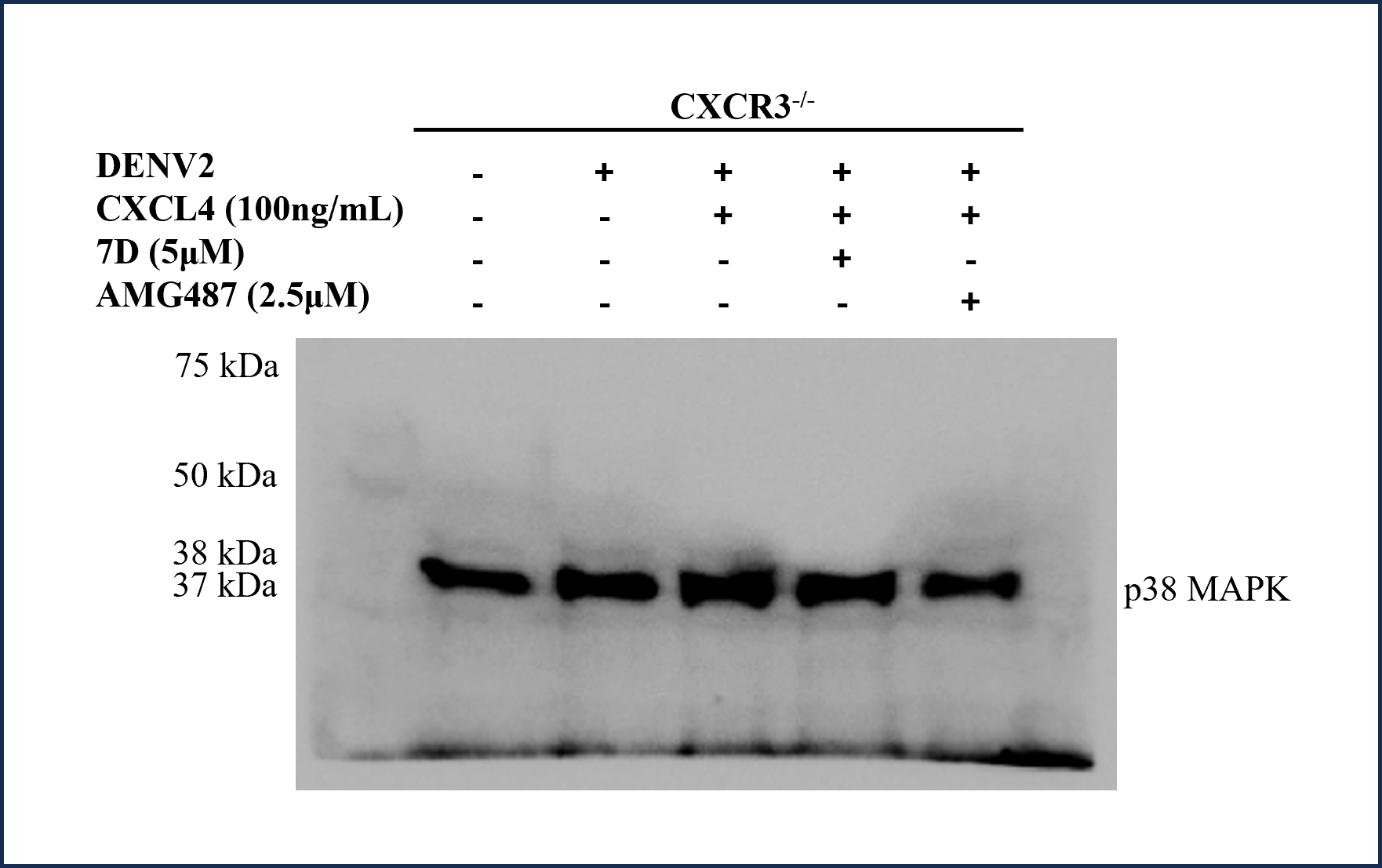

Supplement: Supplementary file 4 — Source data Fig. 2 [file 44321_2024_137_MOESM4_ESM.zip › Fig 2/Fig 2G/Western p38 MAPK.tif]

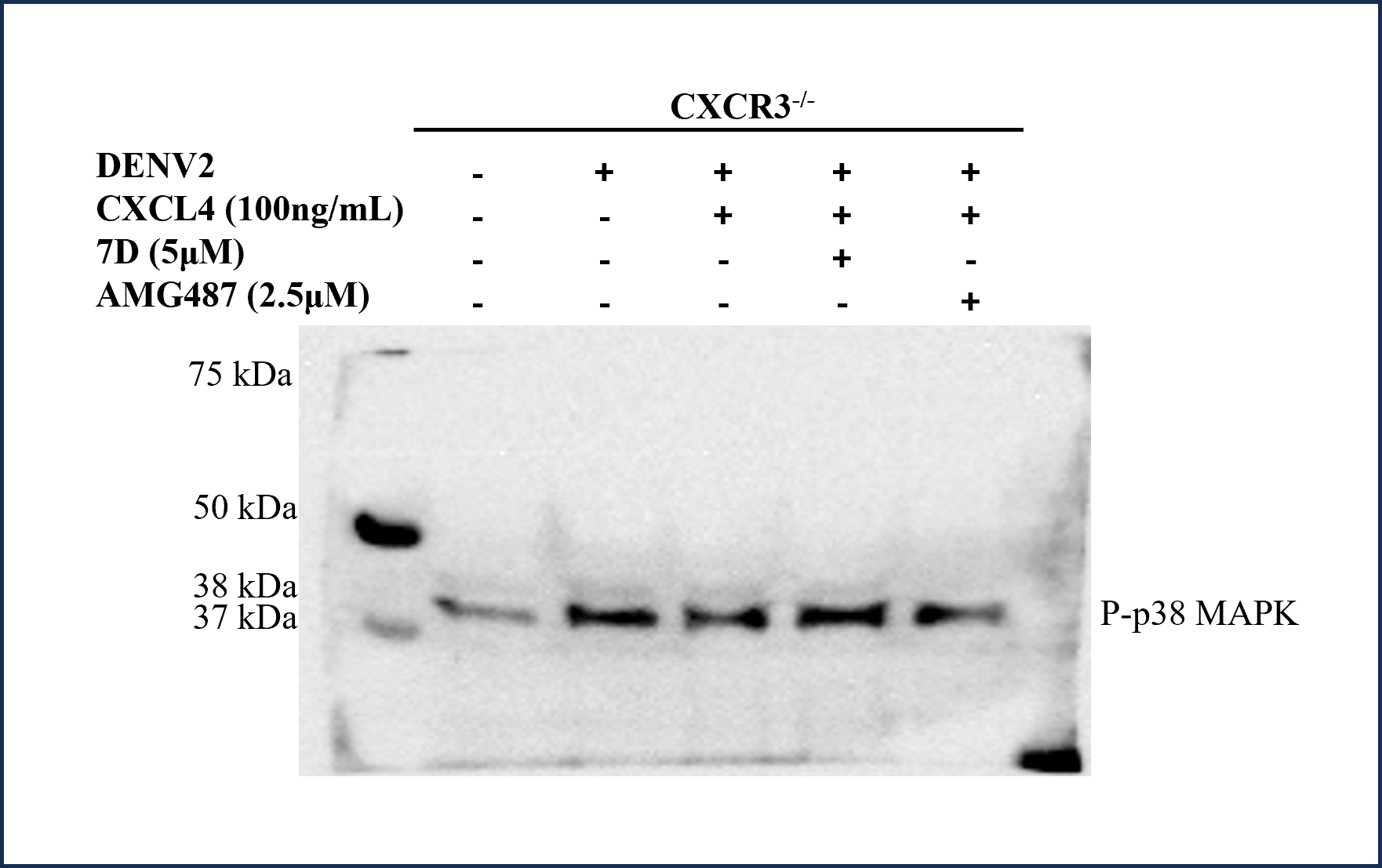

Supplement: Supplementary file 4 — Source data Fig. 2 [file 44321_2024_137_MOESM4_ESM.zip › Fig 2/Fig 2G/Western P-p38 MAPK.tif]

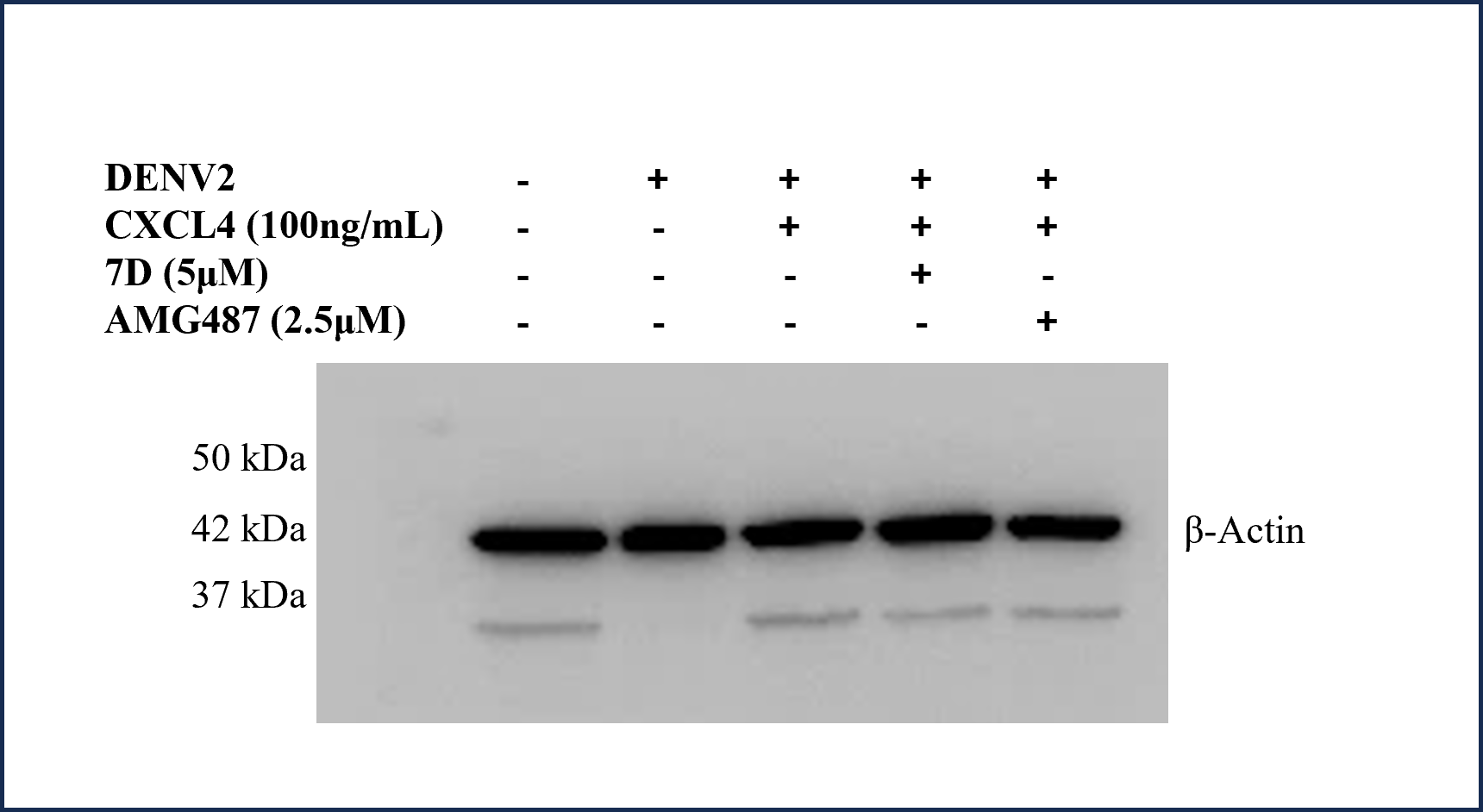

Supplement: Supplementary file 5 — Source data Fig. 3 [file 44321_2024_137_MOESM5_ESM.zip › Fig 3/Fig 3D/Western B-Actin.tif]

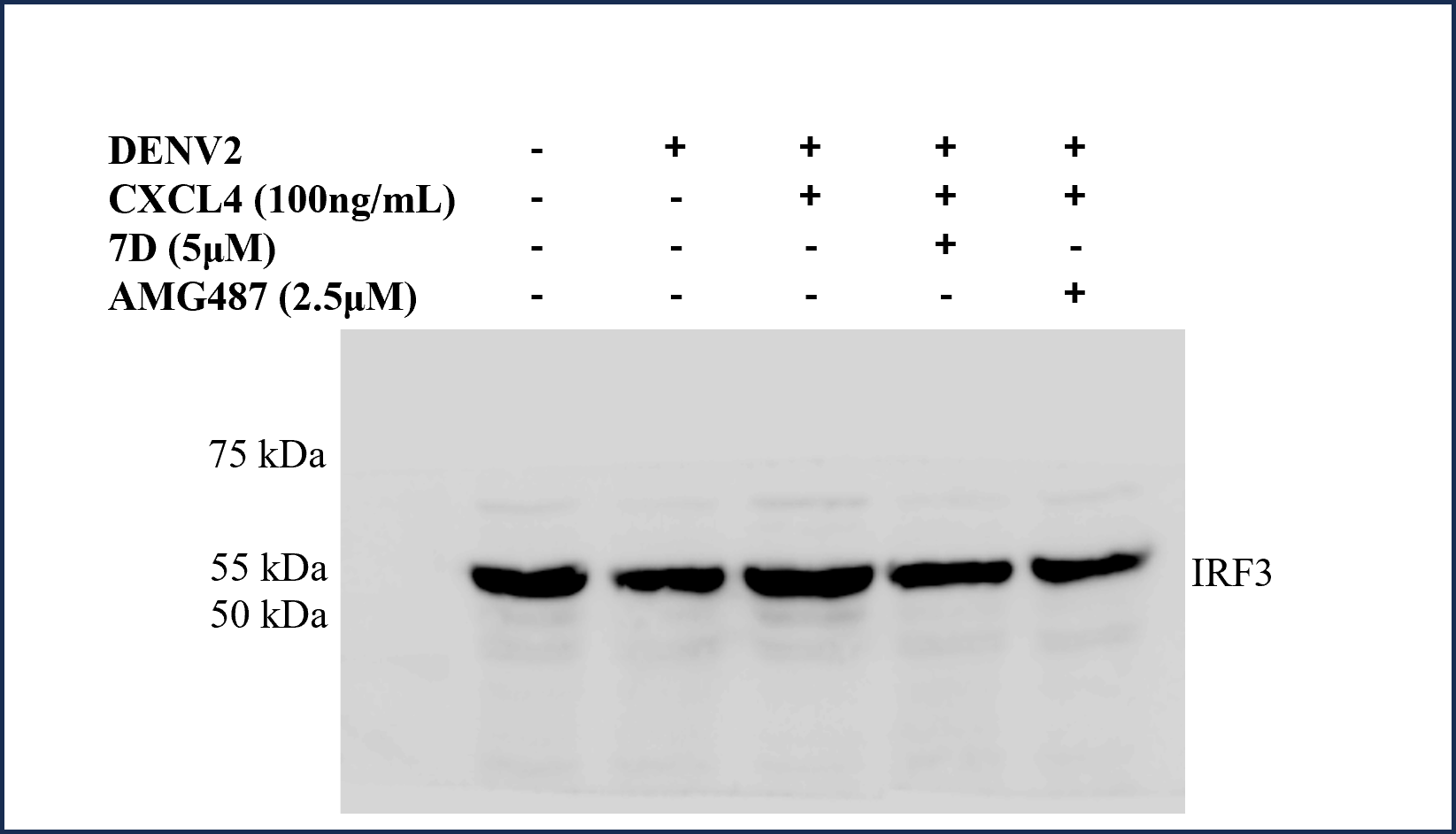

Supplement: Supplementary file 5 — Source data Fig. 3 [file 44321_2024_137_MOESM5_ESM.zip › Fig 3/Fig 3D/Western IRF3.tif]

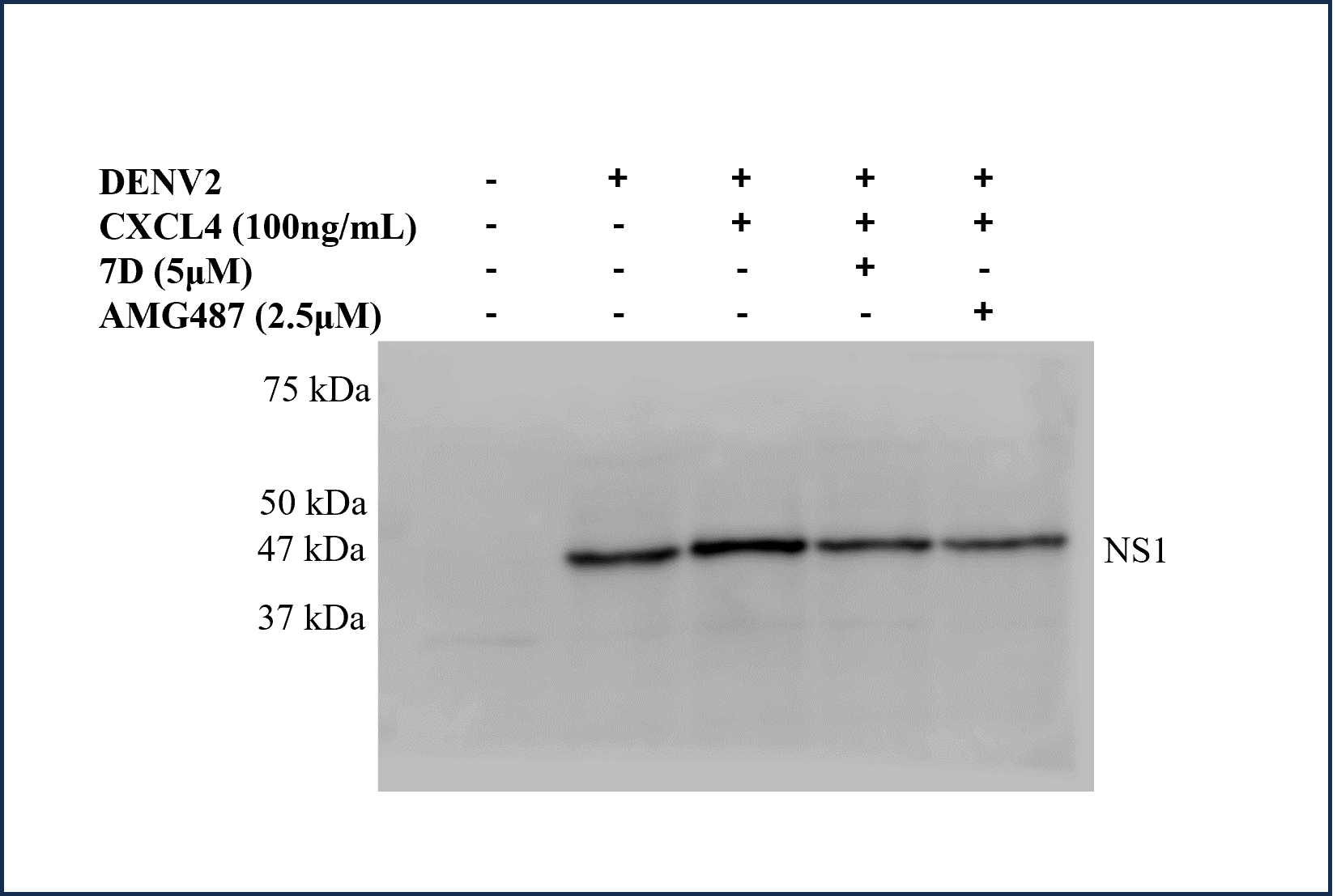

Supplement: Supplementary file 5 — Source data Fig. 3 [file 44321_2024_137_MOESM5_ESM.zip › Fig 3/Fig 3D/Western NS1.tif]

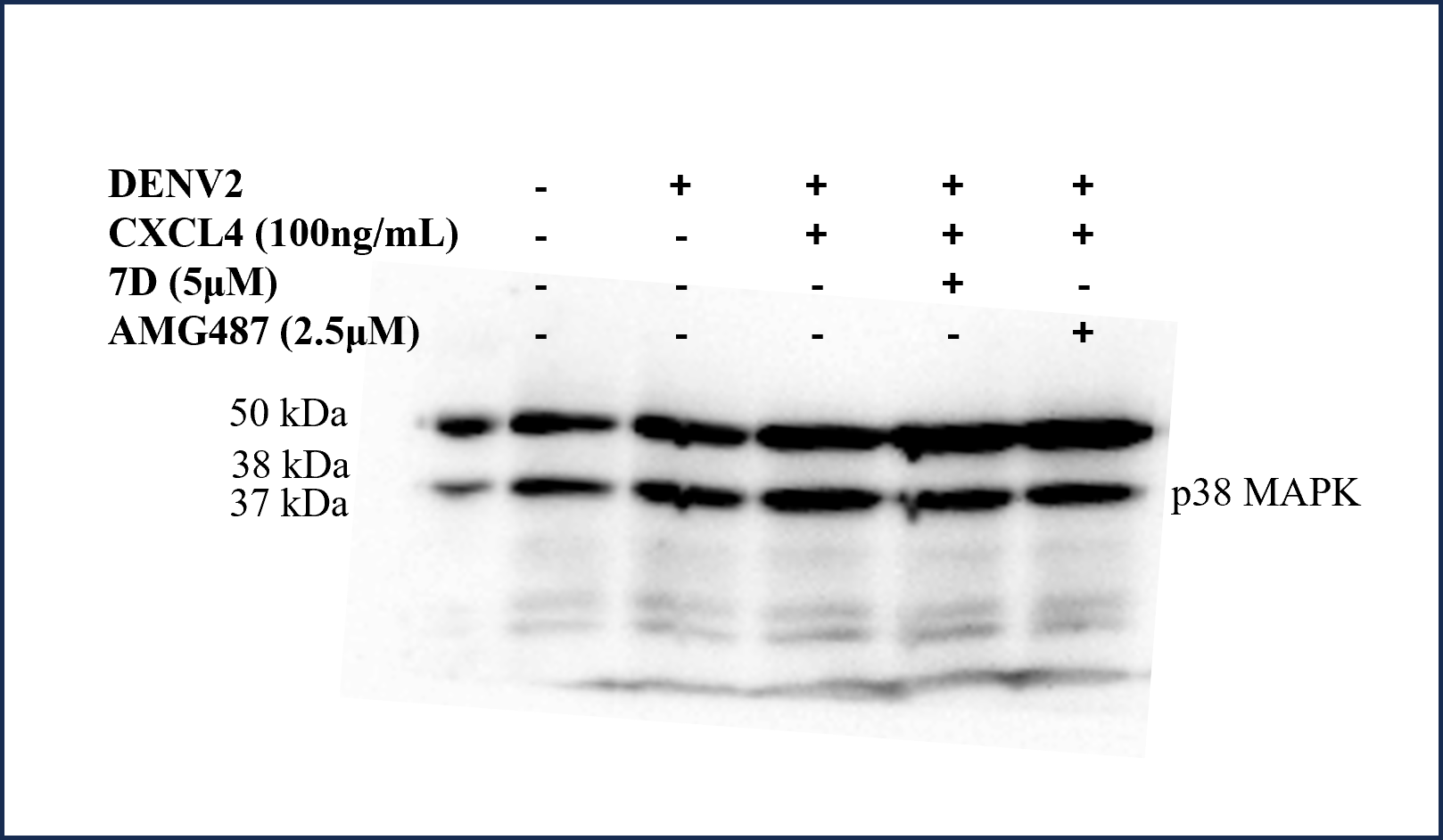

Supplement: Supplementary file 5 — Source data Fig. 3 [file 44321_2024_137_MOESM5_ESM.zip › Fig 3/Fig 3D/Western p38 MAPK.tif]

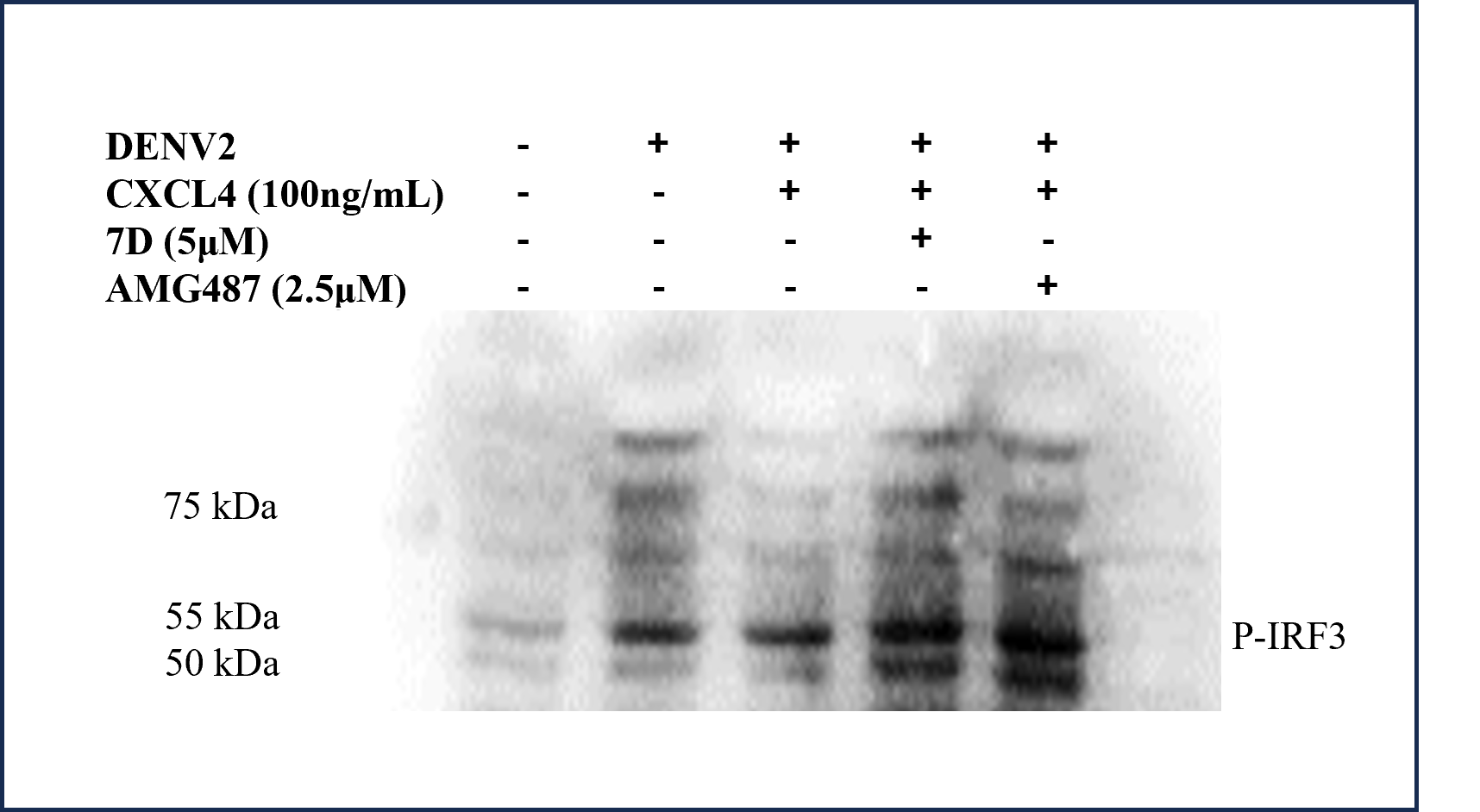

Supplement: Supplementary file 5 — Source data Fig. 3 [file 44321_2024_137_MOESM5_ESM.zip › Fig 3/Fig 3D/Western P-IRF3.tif]

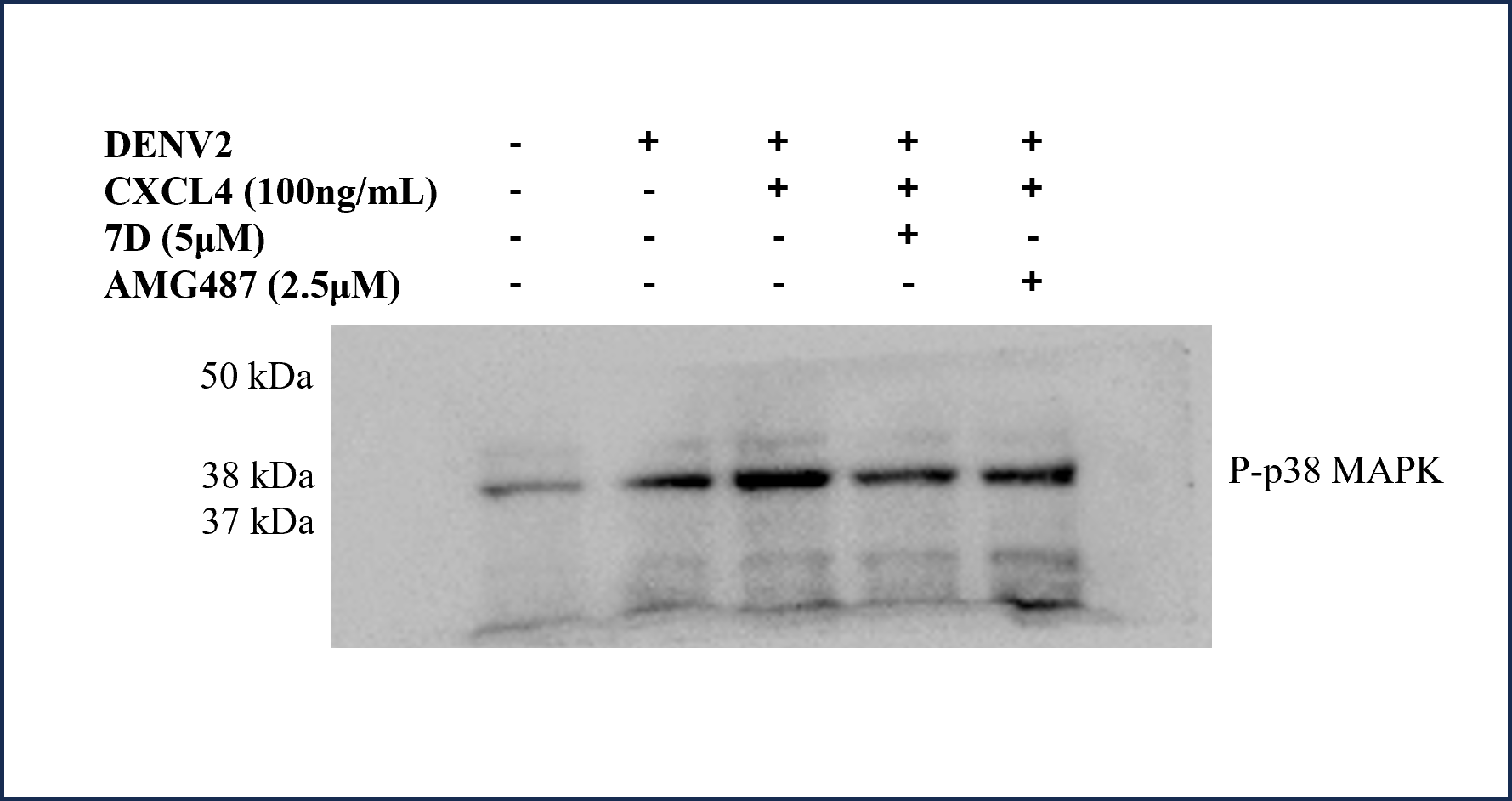

Supplement: Supplementary file 5 — Source data Fig. 3 [file 44321_2024_137_MOESM5_ESM.zip › Fig 3/Fig 3D/Western P-p38 MAPK.tif]

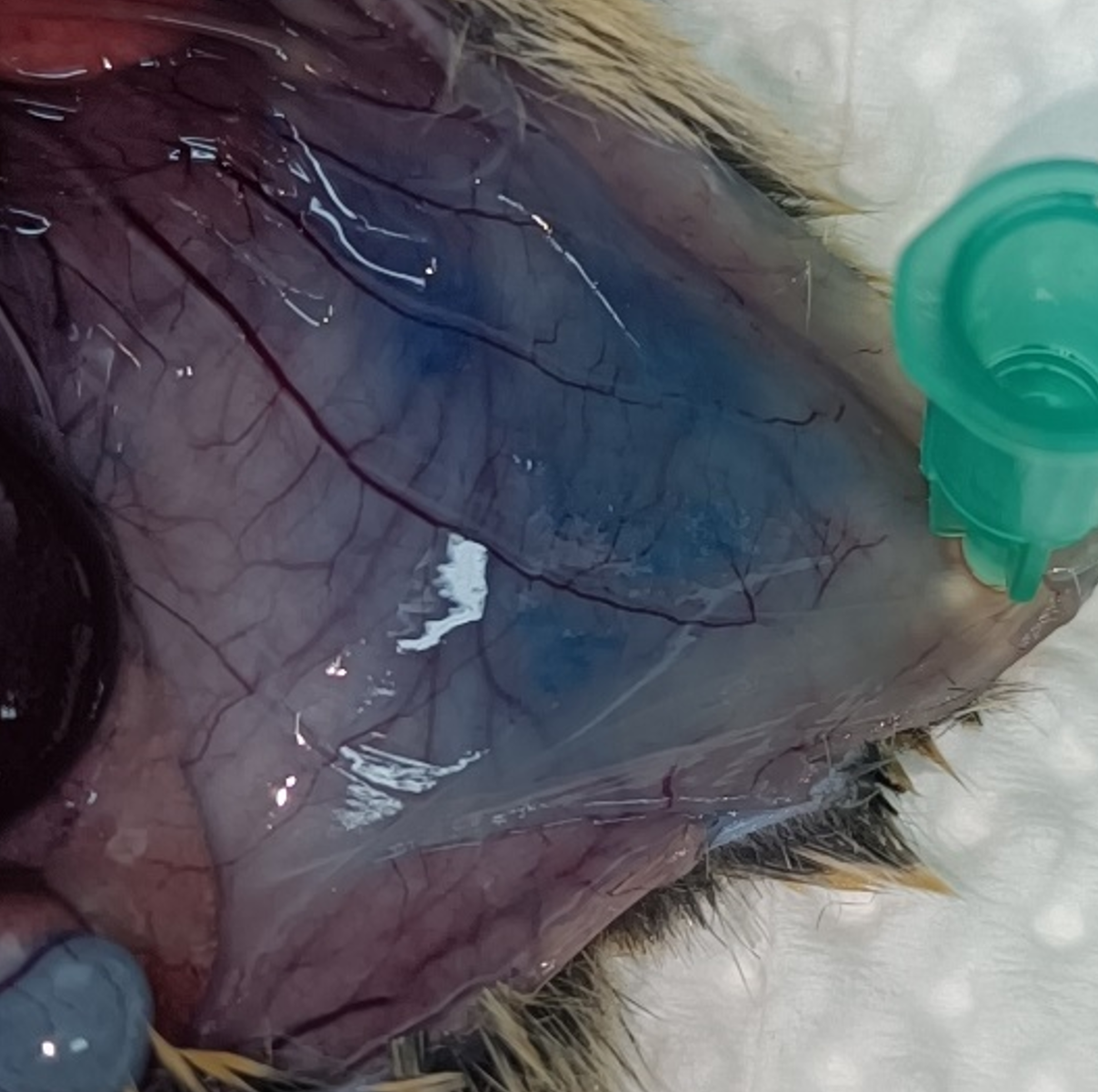

Supplement: Supplementary file 6 — Source data Fig. 4 [file 44321_2024_137_MOESM6_ESM.zip › Fig 4/Fig 4N/Vascular leakage (EB Dye) from peritoneal veins of mice/DENV2 Infected mice.tif]

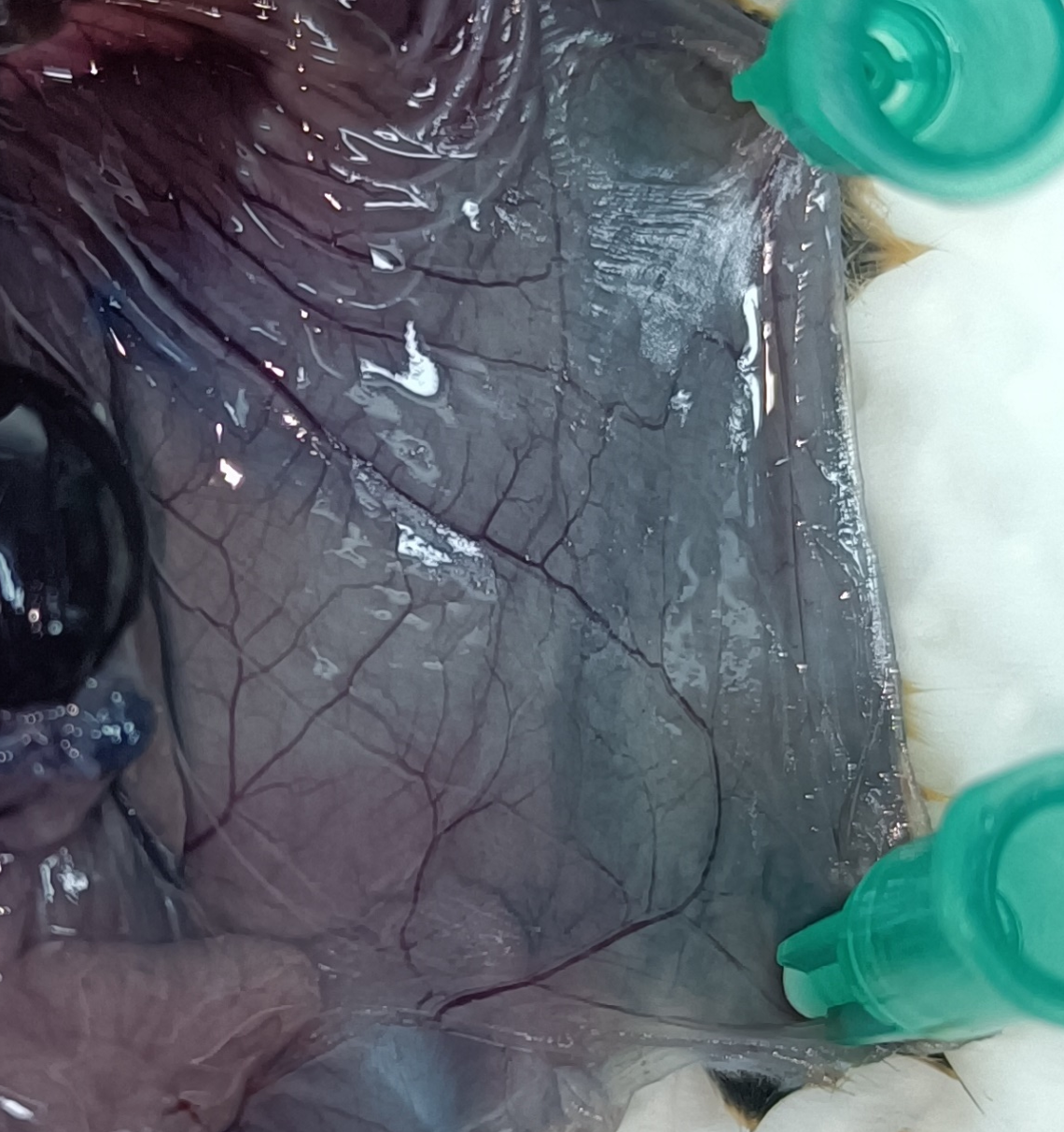

Supplement: Supplementary file 6 — Source data Fig. 4 [file 44321_2024_137_MOESM6_ESM.zip › Fig 4/Fig 4N/Vascular leakage (EB Dye) from peritoneal veins of mice/DENV2+7D Treated mice.tif]

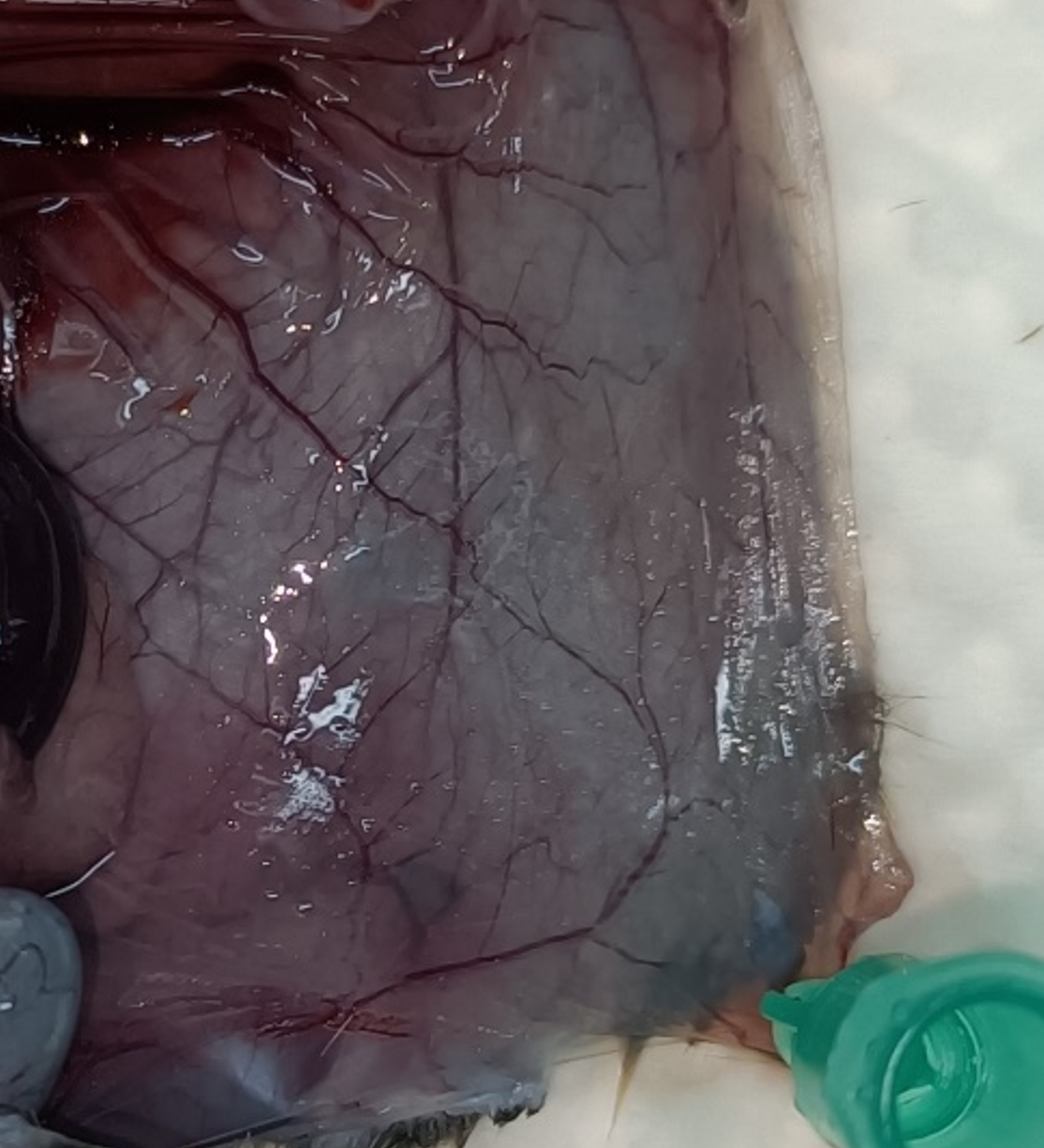

Supplement: Supplementary file 6 — Source data Fig. 4 [file 44321_2024_137_MOESM6_ESM.zip › Fig 4/Fig 4N/Vascular leakage (EB Dye) from peritoneal veins of mice/Vehicle mice.tif]

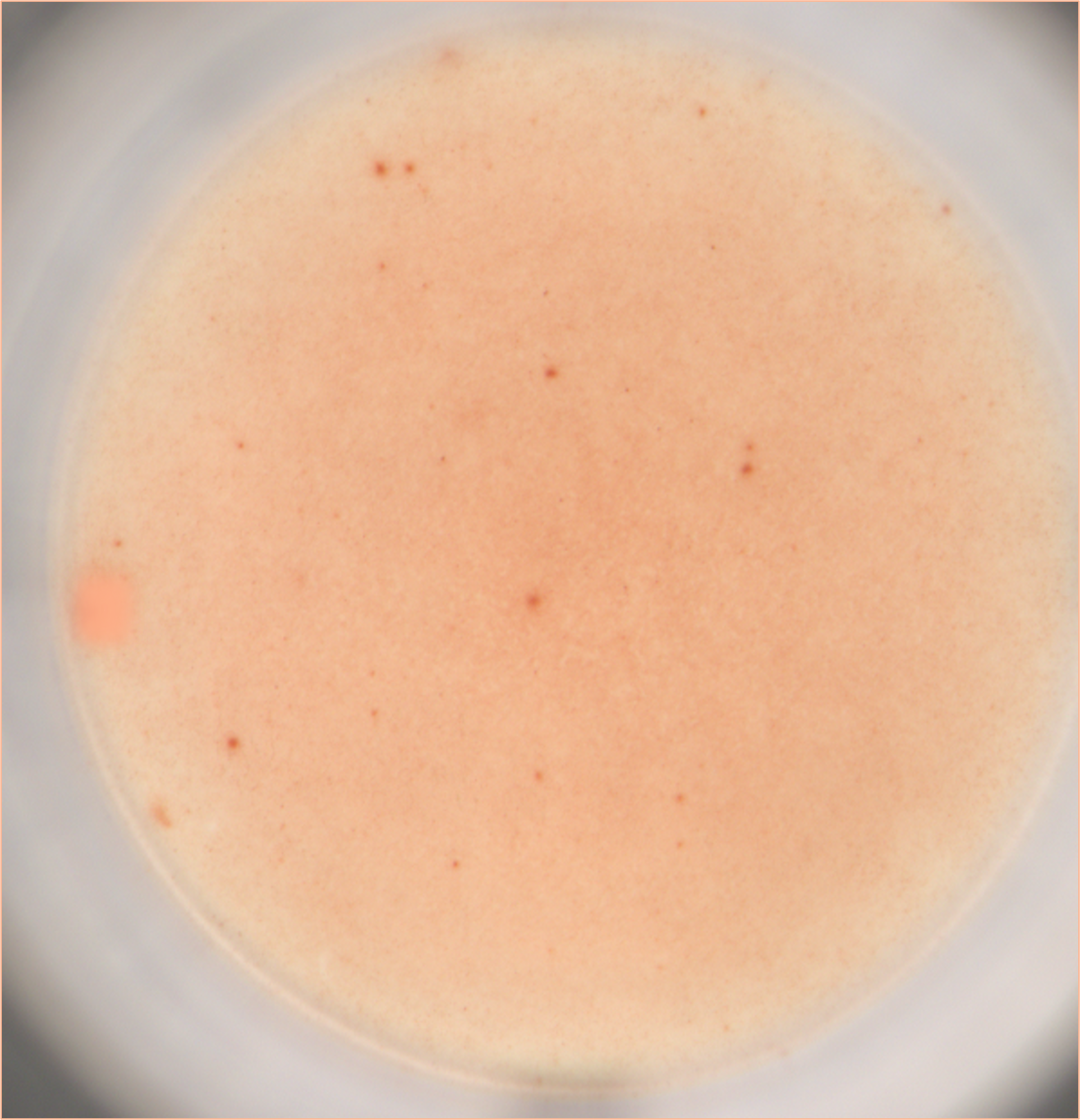

Supplement: Supplementary file 7 — Source data Fig. 5 [file 44321_2024_137_MOESM7_ESM.zip › Fig 5/Fig 5F/Elispot Images/DENV2.tif]

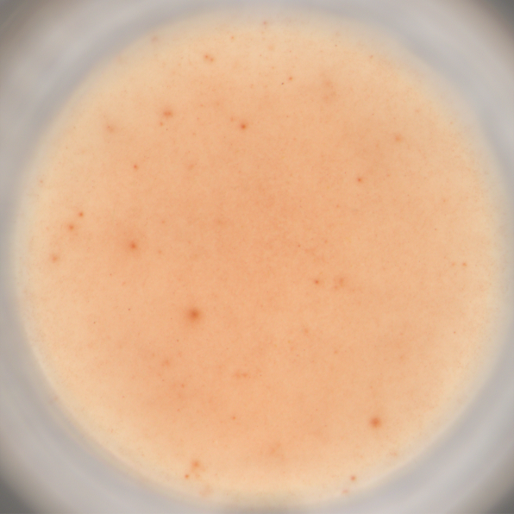

Supplement: Supplementary file 7 — Source data Fig. 5 [file 44321_2024_137_MOESM7_ESM.zip › Fig 5/Fig 5F/Elispot Images/DENV2+7D.tif]

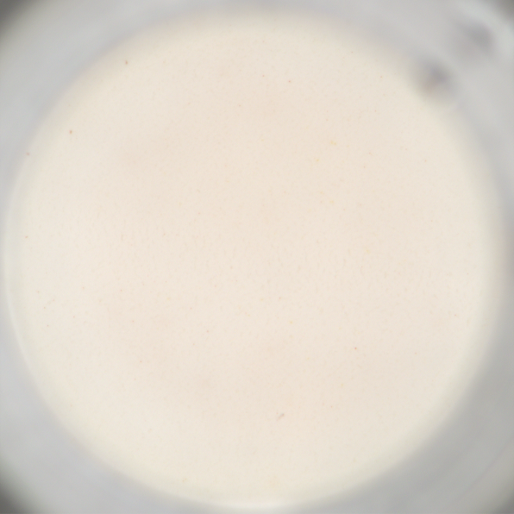

Supplement: Supplementary file 7 — Source data Fig. 5 [file 44321_2024_137_MOESM7_ESM.zip › Fig 5/Fig 5F/Elispot Images/Mock.tif]

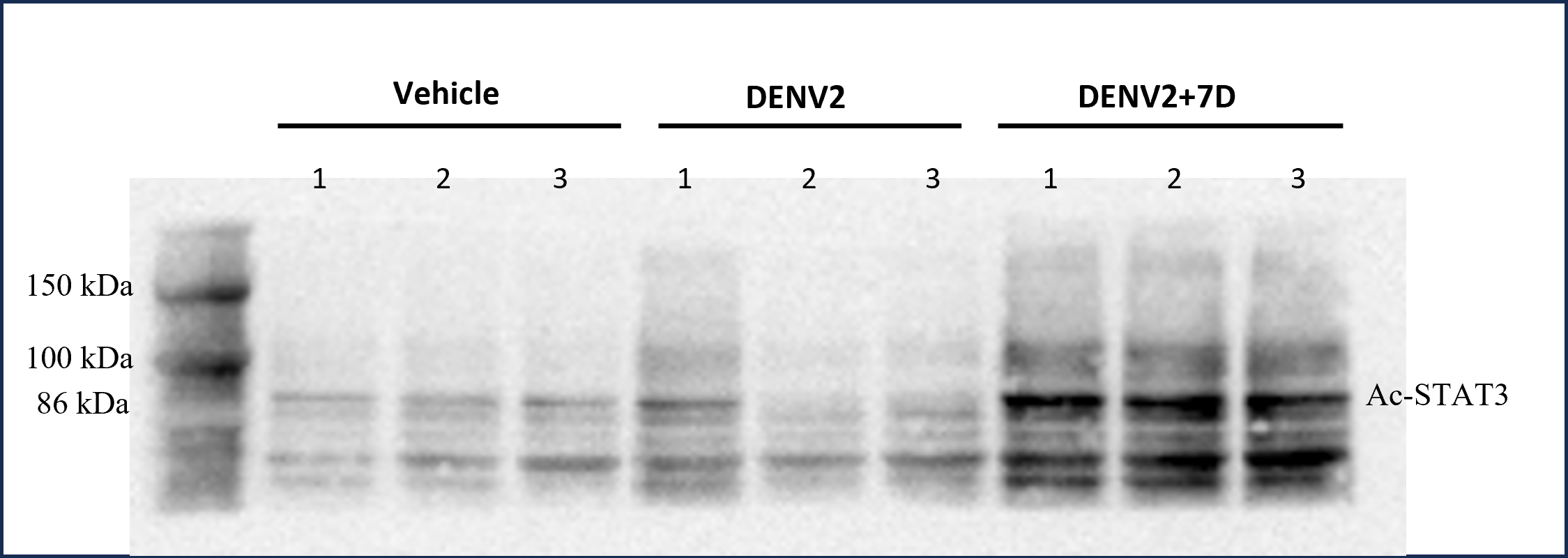

Supplement: Supplementary file 7 — Source data Fig. 5 [file 44321_2024_137_MOESM7_ESM.zip › Fig 5/Fig 5K/Western Ac-STAT3.tif]

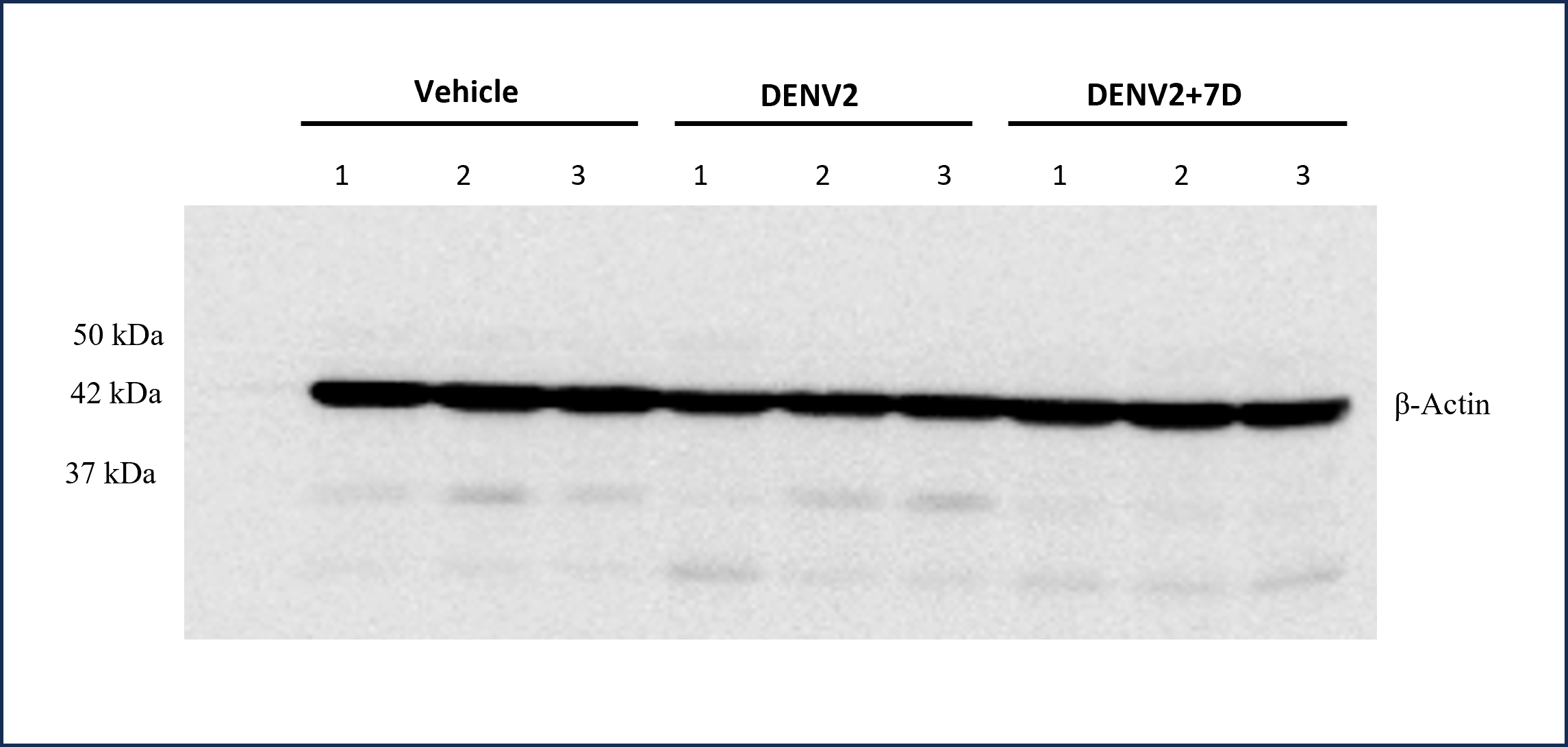

Supplement: Supplementary file 7 — Source data Fig. 5 [file 44321_2024_137_MOESM7_ESM.zip › Fig 5/Fig 5K/Western B-Actin.tif]

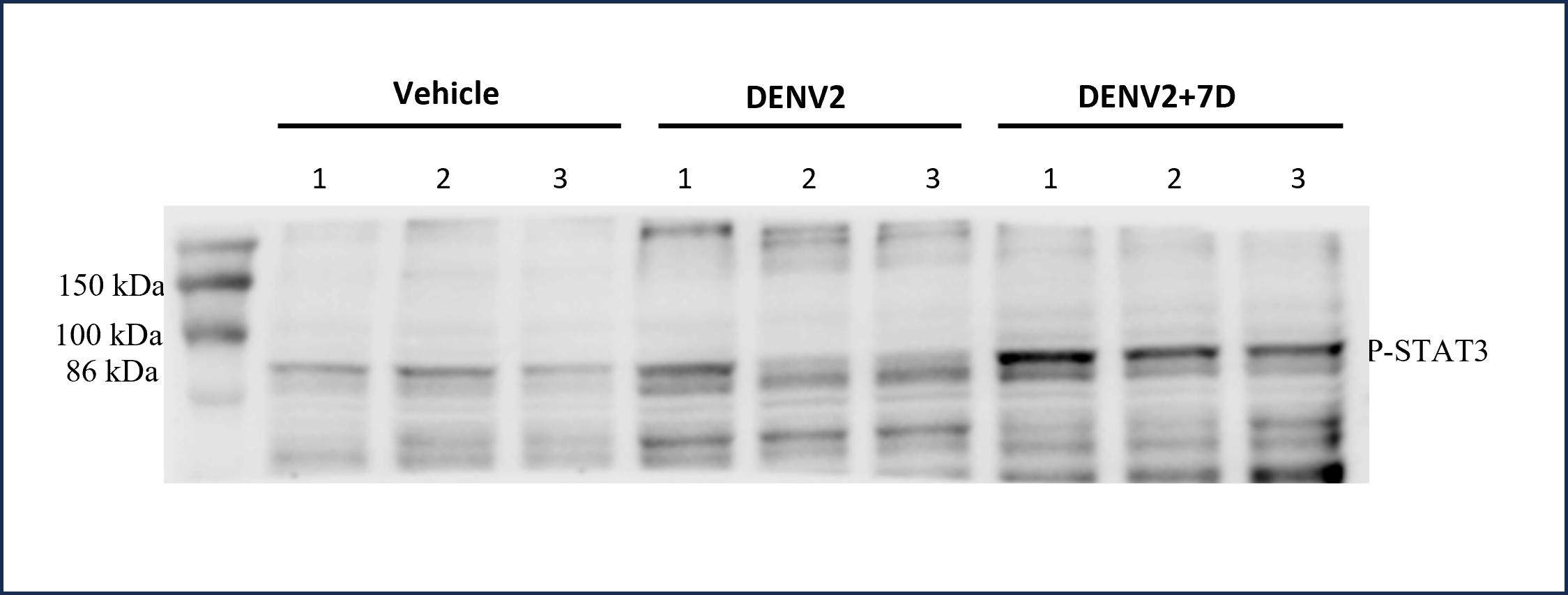

Supplement: Supplementary file 7 — Source data Fig. 5 [file 44321_2024_137_MOESM7_ESM.zip › Fig 5/Fig 5K/Western P-STAT3.tif]

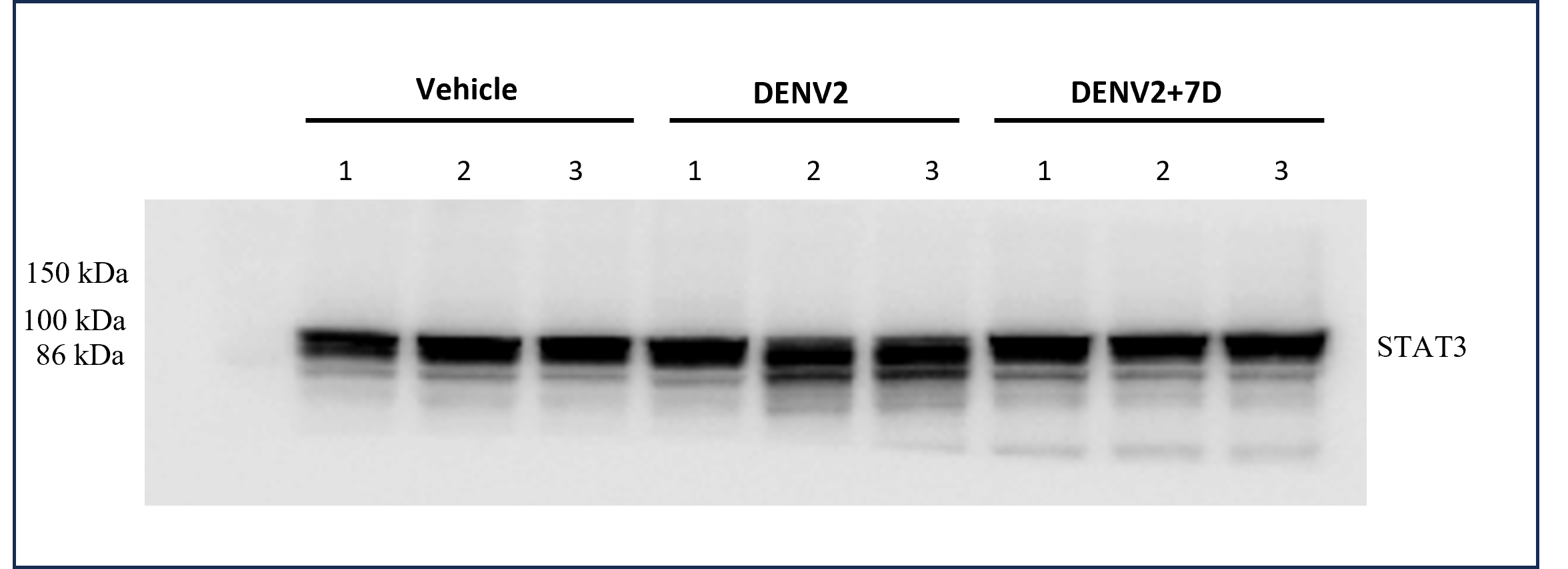

Supplement: Supplementary file 7 — Source data Fig. 5 [file 44321_2024_137_MOESM7_ESM.zip › Fig 5/Fig 5K/Western STAT3.tif]

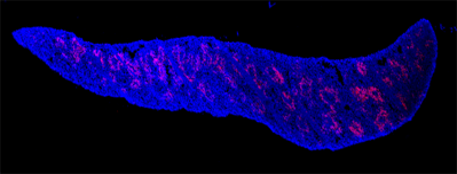

Supplement: Supplementary file 7 — Source data Fig. 5 [file 44321_2024_137_MOESM7_ESM.zip › Fig 5/Fig 5N/Microscopy images for GL7 positive follicles in mice spleen/DENV2 GL7 Staining.tif]

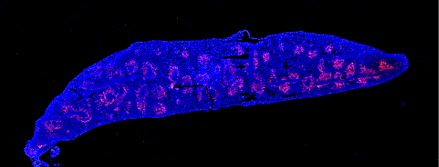

Supplement: Supplementary file 7 — Source data Fig. 5 [file 44321_2024_137_MOESM7_ESM.zip › Fig 5/Fig 5N/Microscopy images for GL7 positive follicles in mice spleen/DENV2+7D GL7 Staining.tif]

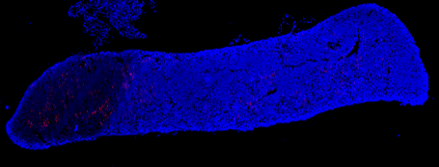

Supplement: Supplementary file 7 — Source data Fig. 5 [file 44321_2024_137_MOESM7_ESM.zip › Fig 5/Fig 5N/Microscopy images for GL7 positive follicles in mice spleen/Vehicle GL7 Staining.tif]
