# Supplementary material for: 7D, a small molecule inhibits dengue infection by increasing interferons and neutralizing-antibodies via CXCL4:CXCR3:p38:IRF3 and Sirt1:STAT3 axes respectively
Source: EMBO Mol Med. 2024 Sep 16;16(10):2376–401. doi: 10.1038/s44321-024-00137-8 (PMC11473809; doi:10.1038/s44321-024-00137-8)
Supplement: Supplementary file 9 — Expanded View Figures [file 44321_2024_137_MOESM9_ESM.pdf]

Expanded View Figures

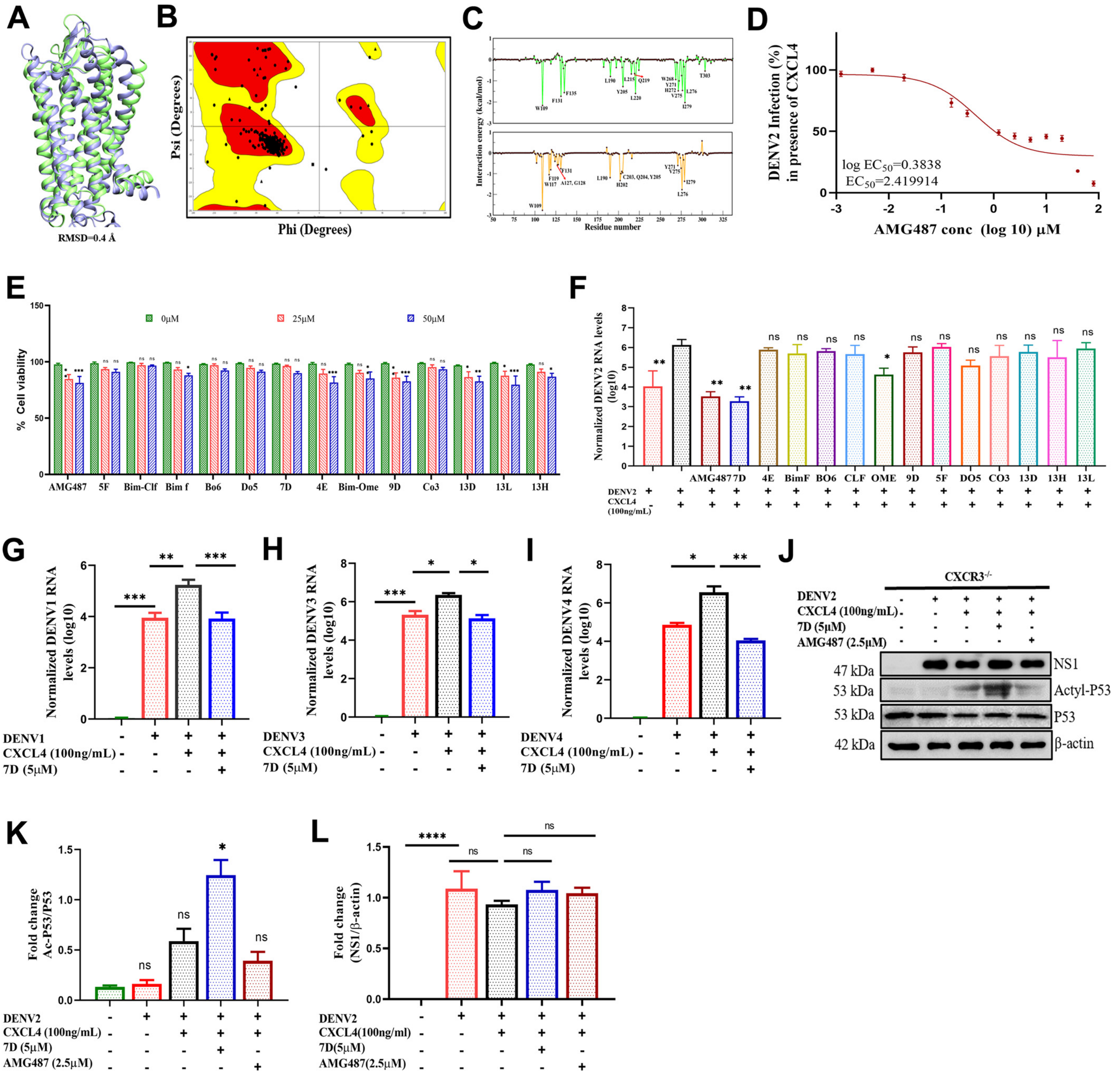

Figure EV1. In silico drug design and viral replication data related to main Fig. 1.

(**A**) Superimposed structures of crystal CCR5 (blue) and model of CXCR3 (green). (**B**) Ramachandran plot of CXCR3 homology model (**C**) CXCR3-7D (green) and CXCR3-AMG487 (yellow) binding site interaction energies of residues. Related data of Fig. 1J,K. (**D**) U937-DC-SIGN cells were infected with MOI ~ 1 of DENV2 (P23085 INDI-60) in presence CXCL4 (100 ng/ml) and various concentrations of AMG487. The EC_{50} value of AMG487 was calculated from the dose-response curve from drug effects on viral genome (measured by qRT-PCR) from independent experiments, $n = 3$. (**E**) Cell viability was measured after treatment with all 13 compounds from in silico hits. U937-DC-SIGN cells were incubated with the compounds for 48 h and cell viability were measured using MTT assay, $n = 3$ independent experiments, two-way ANOVA was used (*P* values: 0.004; 0.0004; 0.01; 0.0002; 0.005; 0.005; 0.0004; 0.02; 0.0019; 0.01; 0.0001; 0.01). (**F**) Effects of 13 compounds (2.5 µM) on DENV2 replication in U937-DC-SIGN cells was measured in presence of CXCL4 (100 ng/ml). Viral genome was quantified using qRT-PCR from independent experiments, n = 3, one-way ANOVA and Kruskal–Wallis test was used (*P* values: 0.008; 0.003; 0.0014; 0.02). (**G–I**) U937-DC-SIGN cells were infected with MOI ~ 1 of (**G**) DENV1 (**H**) DENV3 and (**I**) DENV4 (strain H241) in presence of CXCL4 (100 ng/ml) and 7D (5 µM) and viral genome was quantified using qRT-PCR from independent experiments, $n = 3$, one-way ANOVA and Bonferroni's post-test were used (*P* values: **G**: 0.001; 0.0014; 0.0008, **H**: 0.001; 0.01; 0.02, **I**: 0.01; 0.003). (**J–L**) Western blot was performed for (**J**) NS1, actyl-p53, p53 and β-actin in lysate from $CXCR3^{-/-}$ monocytes from Fig. 2G. Densitometry of (**K**) actyl-p53 and (**L**) NS1 blots, $n = 3$ independent experiments, one-way ANOVA and Bonferroni's post-test were used (*P* value: **K**: 0.01, **L**: 0.0001). Data information: (**D–I**, **K**, **L**) Data are mean ± SEM, *$P < 0.05$, **$P < 0.01$, ***$P < 0.001$, ****$P < 0.0001$, ns=non-significant.

 © The Author(s)

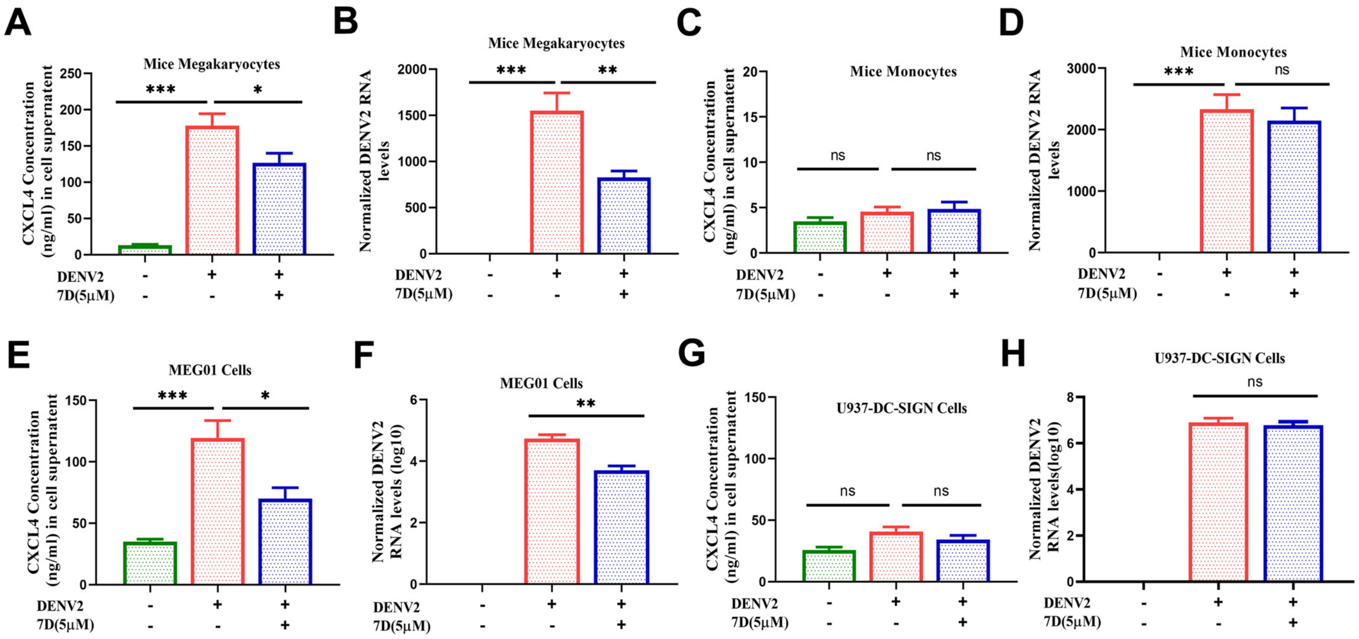

Figure EV2. Effects of 7D on DENV2 replication on megakaryocytes in vitro.

(**A**, **B**) Megakaryocytes isolated from mice bone marrow, and infected with DENV2 (MOI ~ 1) for 24 h with and without 7D. Culture supernatant was used for measuring (**A**) CXCL4 levels using ELISA, and (**B**) cell pallet was used for detecting viral genome using qRT PCR, $n = 3$ independent experiments for both (P values: **A**: 0.0002; 0.04, **B**: 0.0002; 0.008). (**C**, **D**) Similar experiment was performed in monocytes (as CXCL4 non-producing cells) isolated from whole blood of these mice. (**C**) CXCL4 and (**D**) DENV2 genome were detected, $n = 3$ independent experiments for both assays (P value: 0.0002). A similar experiment was performed in (**E**, **F**) human MEG-01 cell line (CXCL4 producing cells), $n = 3$ independent experiments (P values: **E**: 0.0008; 0.01, **F**: 0.006) and (**G**, **H**) U937-DC-SIGN cell line (CXCL4 non-producing cells), $n = 3$ independent experiments for above assays. One-way ANOVA and Bonferroni's post-test were used for data analysis. Data information: (**A–E**, **G**) One-way ANOVA and Bonferroni's post-test were used for data analysis. Data are mean ± SEM. (**F**, **H**) Student's t-test was used. Data are mean ± SEM. *$P < 0.05$, **$P < 0.01$, ***$P < 0.001$, ns=non-significant.

© The Author(s)

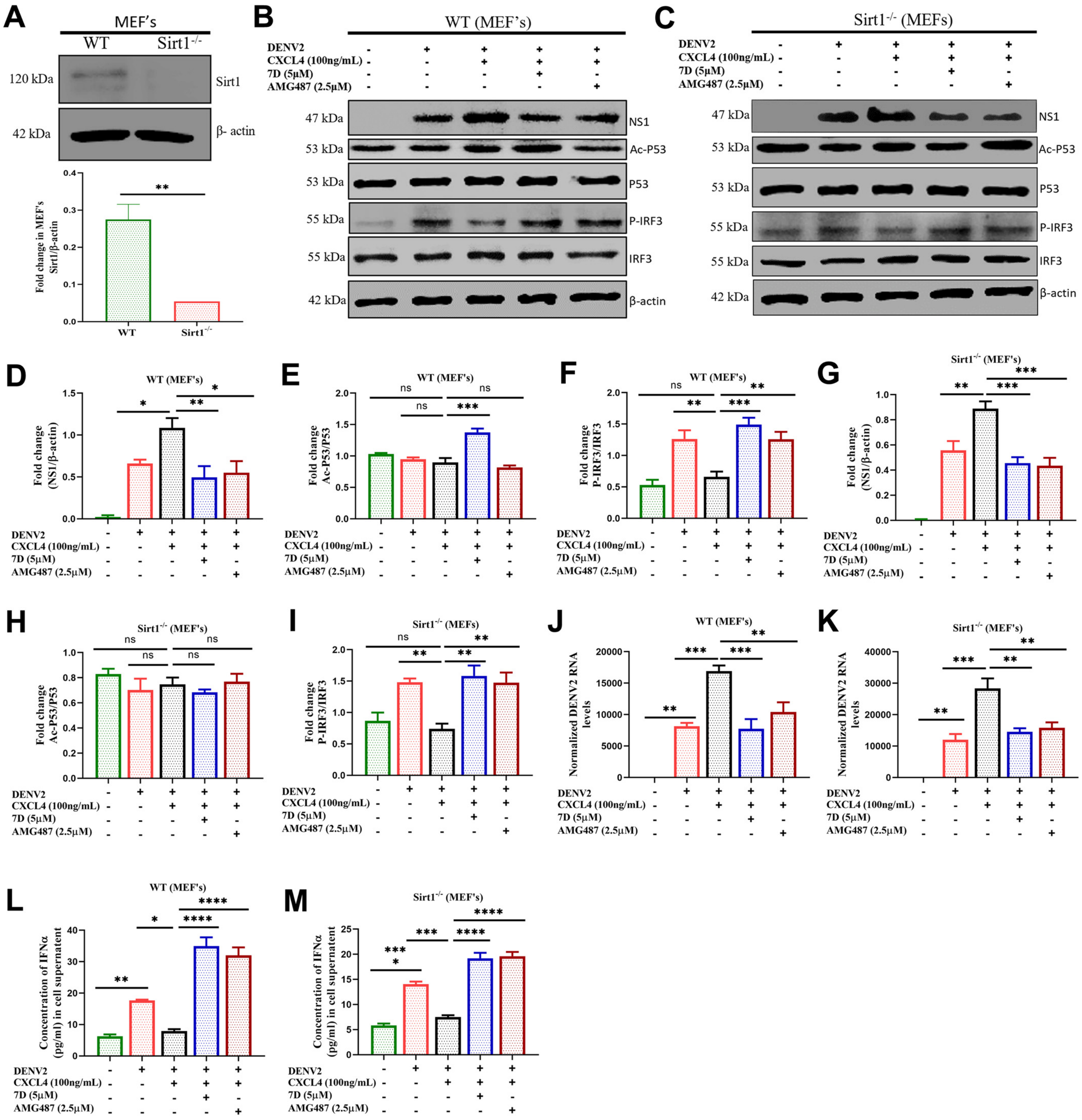

Figure EV3. Effects of 7D on DENV2 replication in $Sirt1^{-/-}$ MEFs in vitro.

(**A**) Sirtuin-1 (Sirt1) expression in $Sirt1^{-/-}$ and WT mouse embryonic fibroblasts (MEFs) in western blot assay. Densitometry analysis independent experiments $n = 3$, Student's t test was used (P value: 0.005). Above cells were infected with DENV2 and treated with 7D as mentioned for U937-DC-SIGN in Fig. 1F. After experiment, (**B**) WT and (**C**) $Sirt1^{-/-}$ cells were used for western blot analysis for NS1, Ac-P53 and P-IRF3. (**D–I**) Densitometry data from, $n = 3$ independent experiments, one-way ANOVA and Bonferroni's post-test were used (P values: **D**: 0.04; 0.007; 0.01, **E**: 0.001, **F**: 0.009; 0.0002; 0.009, **G**: 0.005; 0.0008; 0.0006, **I**: 0.006; 0.002; 0.007). (**J, K**) Viral genome was quantified in WT and $Sirt1^{-/-}$ MEFs pellet using qRT-PCR, $n = 3$ independent experiments, one-way ANOVA and Bonferroni's post-test were used (P values: **J**: 0.0013; 0.0007; 0.0005; 0.006, **K**: 0.004; 0.0004; 0.002; 0.003). (**L, M**) IFNα level was measured using ELISA from cell supernatant of above experiments, $n = 3$ independent experiments, one-way ANOVA and Bonferroni's post-test were used (P values: **L**: 0.003; 0.01; 0.0001; 0.0001, **M**: 0.0001; 0.0002; 0.0001; 0.0001). Data information: (**A**, **D–M**) Data are mean ± SEM, $^*P < 0.05$, $^{**}P < 0.01$, $^{***}P < 0.001$, $^{****}P < 0.0001$, ns=non-significant.

 © The Author(s)

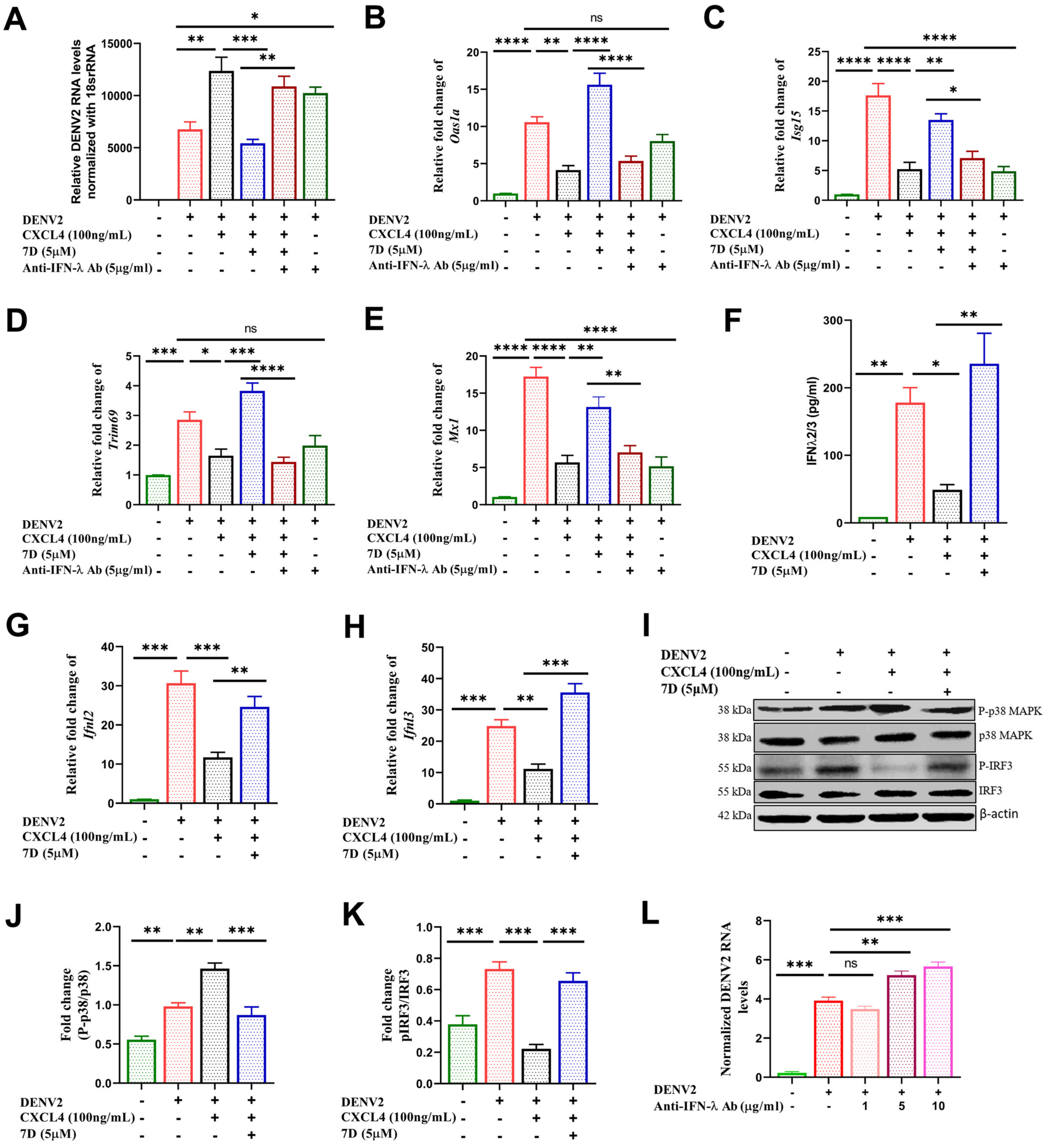
A
Relative DENV2 RNA levels normalized with 18srRNA
B
Relative fold change of Oas1a
C
Relative fold change of Isg15
D
Relative fold change of Trim69
E
Relative fold change of Mx1
F
IFNλ2/3 (pg/ml)
G
Relative fold change of Ifnl2
H
Relative fold change of Ifnl3
I
DENV2
CXCL4 (100ng/mL)
7D (5μM)
38 kDa
P-p38 MAPK
p38 MAPK
55 kDa
P-IRF3
IRF3
42 kDa
β-actin
J
Fold change (P-p38/p38)
K
Fold change pIRF3/IRF3
L
Normalized DENV2 RNA levels
DENV2
CXCL4 (100ng/mL)
7D (5μM)
Anti-IFN-λ Ab (5μg/ml)
Anti-IFN-λ Ab (μg/ml)
ns

Figure EV4. Effects of 7D on DENV2 replication in BMDMs of AG129 mice.

DENV2 infection experiment was performed in bone marrow derived macrophages (BMDMs), isolated from AG129 mice, in presence of 7D. A blocking antibody to IFNλ2/3 (5 µg/ml, standardization of working concentration is mentioned in Fig. EV4L below) was used to investigate the effects of type-III IFN. (**A**) Viral genome was quantified in cell pellets using qRT-PCR, $n = 3$ independent experiments (P values: 0.0011; 0.0002; 0.03; 0.0014). (**B–E**) Relative gene expressions of interferon-stimulated genes (ISGs), (**B**) Oas1a, (**C**) Isg15, (**D**) Trim69, and (**E**) Mx1 were measured from above experiments, $n = 3$ independent experiments, (P values: **B**: 0.0001; 0.0013; 0.0001; 0.0001, **C**: 0.0001; 0.0001; 0.0012; 0.01; 0.0001, **D**: 0.0006; 0.01; 0.0008; 0.0001, **E**: 0.0001; 0.0001; 0.0014; 0.009; 0.0001). (**F**) IFN λ2/3 levels were measured in the supernatant of these cells using ELISA, $n = 3$ independent experiments, (P values: 0.004; 0.02; 0.002). (**G**, **H**) Relative gene expressions of *ifnl2* and *ifnl3* were quantified from the above cell pellets, $n = 3$ independent experiments, (P values: **G**: 0.001; 0.001; 0.008, **H**: 0.001; 0.003; 0.001). (**I–K**) Western blot analysis for P-p38:p38 and P-IRF3:IRF3. (**J**, **K**) Densitometry data from, $n = 3$ independent experiments, (P values: **J**: 0.007; 0.003; 0.001, **K**: 0.0006; 0.0002; 0.0003). (**L**) Concentration-dependent effect of blocking antibody against type-III IFN was tested on viral replication. DENV2 mRNA was quantified from pellets of DENV2-infected U937-DC-SIGN cells in presence of increasing concentration (1, 5 and 10 µg/ml) of blocking antibody against IFN-λ2/3 Ab, $n = 3$ independent experiments, (P values: 0.001; 0.002; 0.0002). One-way ANOVA and Bonferroni's post-test were used for above analysis. Data information: (**A–H**, **J–L**) One-way ANOVA and Bonferroni's post-test were used. Data are mean ± SEM, $^*P < 0.05$, $^{**}P < 0.01$, $^{***}P < 0.001$, $^{****}P < 0.0001$ and ns=non-significant.

 © The Author(s)

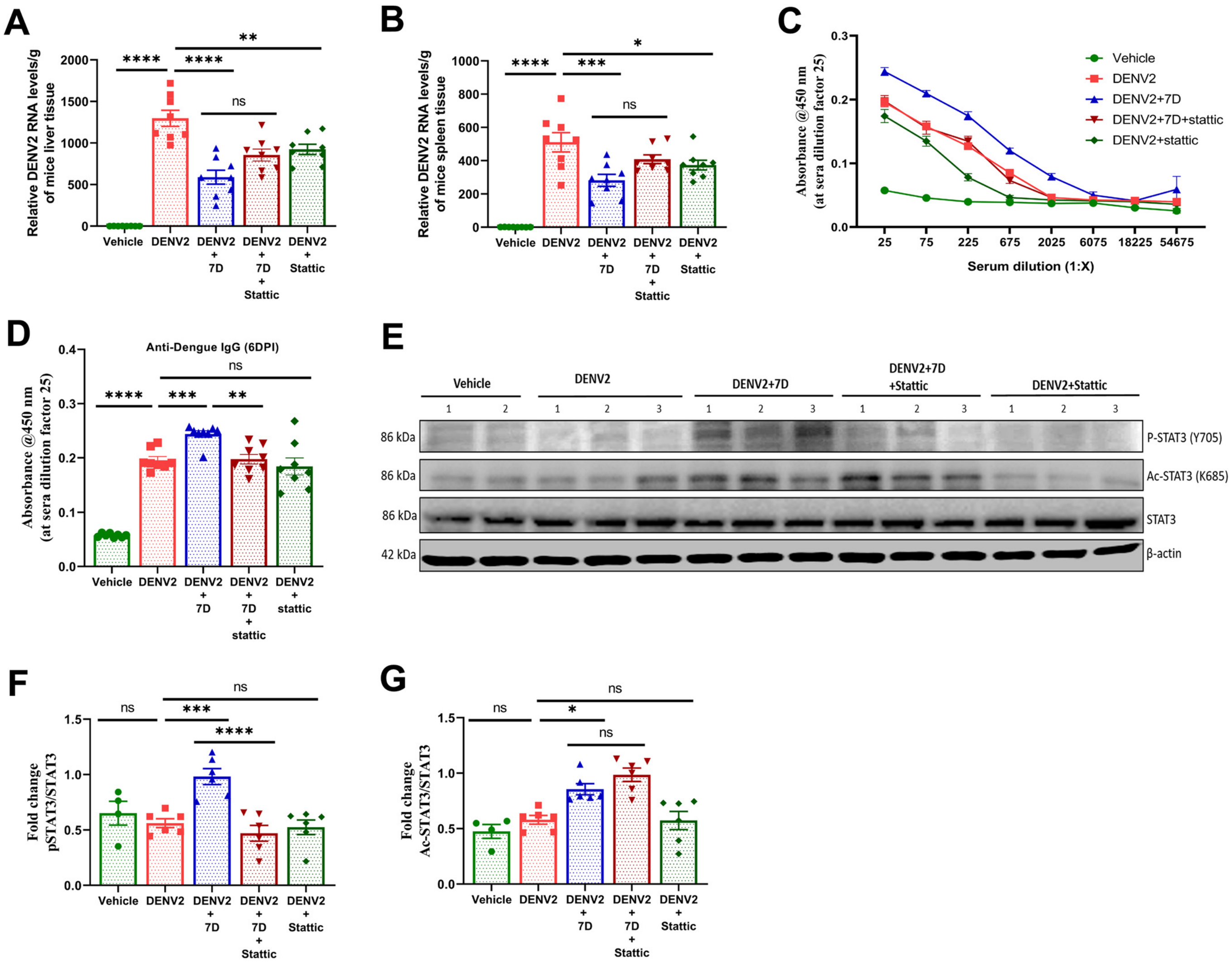

Figure EV5. Effect of STAT3-inhibitor on 7D-mediated antibody generation in DENV2-infected AG129 mice.

As mentioned in the Fig. 4A, a similar experiment was performed in AG129 mice treated with 7D and STAT3-inhibitor Stattic (10 mg/kg/body weight, referred concentration from vendor's manual). (**A**, **B**) DENV2 viral RNA was quantified in liver and spleen respectively using qRT-PCR, $n = 8$ mice per group, (*P* values: **A**: 0.0001; 0.0001; 0.003, **B**: 0.0001; 0.0003; 0.04). (**C**, **D**) Anti-dengue IgG was measured from the serum of mice using ELISA, $n = 8$, (*P* values: 0.0001; 0.0005; 0.003). (**E**–**G**) Ac-STAT3 and P-STAT3 were measured in spleenocytes by western blot and normalized with total STAT3. Densitometry of the above blots, $n = 4$ vehicle, $n = 6$ mice per group (*P* values: **F**: 0.0008; 0.0001, **G**: 0.01). One-way ANOVA and Bonferroni's post-test were used for all above analysis. Data information: (**A**–**D**, **F**, **G**) One-way ANOVA and Bonferroni's post-test were used. Data are mean ± SEM, $^*P < 0.05$, $^{**}P < 0.01$, $^{***}P < 0.001$, $^{****}P < 0.0001$, ns=non-significant.

© The Author(s)

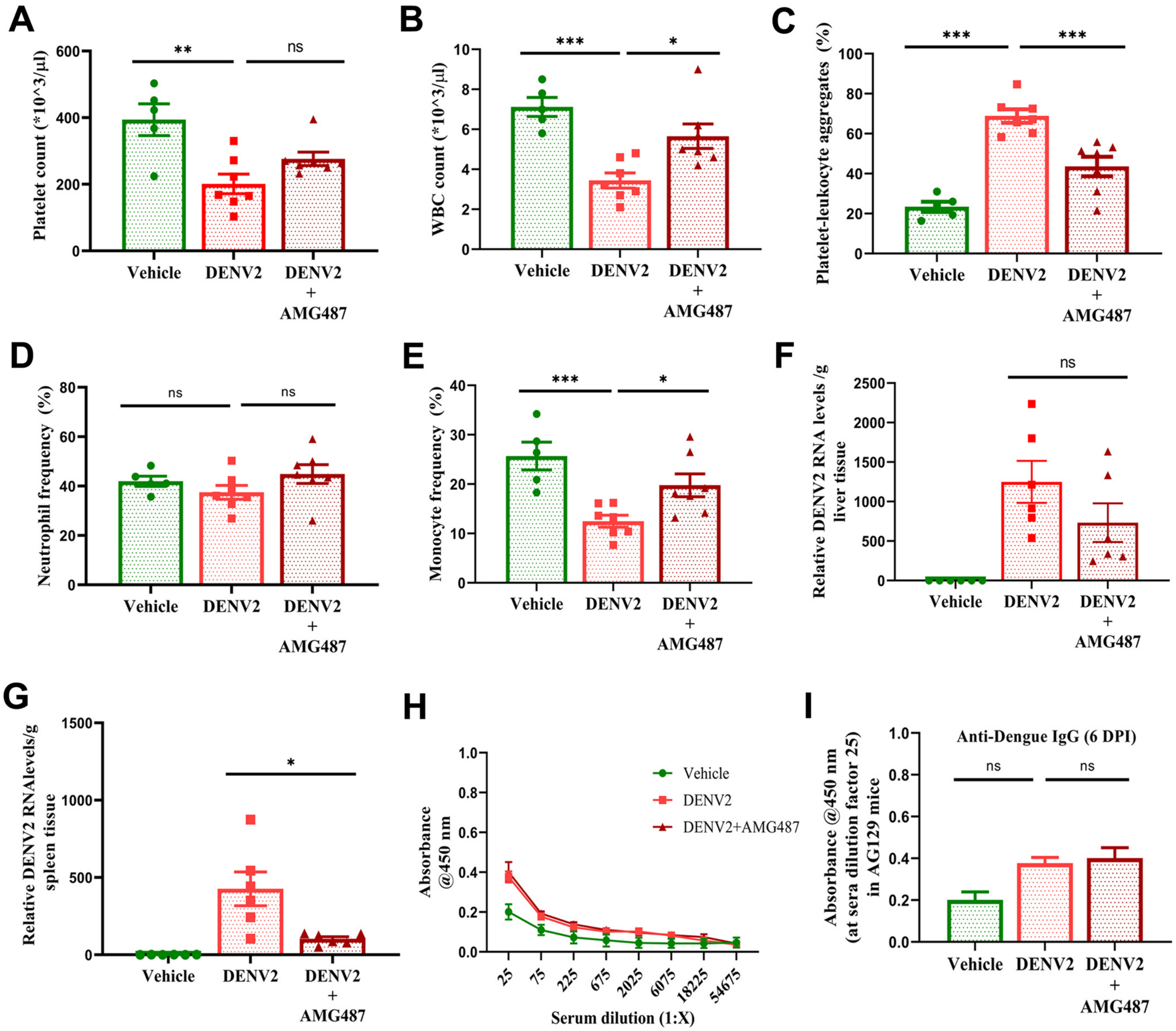

Figure EV6. Effects of AMG487 on DENV2 infection in AG129 mice.

AG129 mice were infected with DENV2 and treated with AMG487 (8 mg/kg body weight, referred concentration from our previous work). (**A**) Platelets and (**B**) WBCs were counted in whole blood using hematology analyzer. (**C**) Platelet-leukocyte aggregates, (**D**) neutrophils and (**E**) monocytes percentage were measured using flow cytometry, $n = 5$ vehicle, $n = 7$ mice per group, one-way ANOVA and Bonferroni's post-test were used for above analysis, (P values: **A**: 0.0015; **B**: 0.0003; 0.01; **C**: 0.001; 0.0005; **E**: 0.001; 0.03). (**F**, **G**) DENV2 genome was quantified by qRT-PCR in (**F**) liver and (**G**) spleen, $n = 6$ mice per group and Mann–Whitney U test was used, (P value: 0.01). (**H**, **I**) IgG against DENV2 antigen was measured in mice plasma, ($n = 6$ per group and one-way ANOVA and Kruskal–Wallis test was used). Sera dilution factor 25 at OD_{450nm}. Data information: (**A**–**E**, **I**) Data are mean ± SEM, and (**F**, **G**) data are median ± IQR, $^{*}P < 0.05$, $^{**}P < 0.01$, $^{***}P < 0.001$, ns=non-significant.

© The Author(s)
